# Piezoionic copolymer ionogel with tunable hydrogen bond enables epidermal blood pressure analyzer

**DOI:** 10.1126/sciadv.aee4501

**Published:** 2026-09-11

**Authors:** Xuejie Liu, Zheng Gong, Zhiheng Zeng, Kai Yang, Bo Wu, Bolong Li, Derek Ho

**Affiliations:** ^1^ Department of Materials Science and Engineering, City University of Hong Kong, Kowloon 999077, Hong Kong, China.; ^2^ Hong Kong Centre for Cerebro-cardiovascular Health Engineering, N.T. 999077, Hong Kong, China.; ^3^ Department of Electrical Engineering, City University of Hong Kong, Kowloon 999077, Hong Kong, China.; ^4^ Institute of Digital Medicine, City University of Hong Kong, Kowloon 999077, Hong Kong, China.

## Abstract

Self-powered sensors that directly convert mechanical stimuli into electrical signals hold promise for next-generation bio-integrated electronics, but their practical translation is hindered by low electrical output and poor mechanical properties. Herein, we present an engineered copolymer ionogel featuring piezoionic amplification and enhanced mechanical properties through optimization of the hydrogen bond network by inserting polyurea chains into poly(vinyl alcohol) (PVA). The optimized copolymer ionogel achieves a voltage output of 20 millivolts, which is 10 folds higher than conventional PVA ionogels. Exceptional stretchability (1510%), toughness (23.1 megajoules per cubic meter), and durability are also achieved. Integrated into a wearable epidermal sensor with a machine learning algorithm, the copolymer ionogel enables cuffless, noninvasive, and continuous blood pressure monitoring, achieving mean absolute errors of 5.9 millimeters of mmHg (mmHg; systolic) and 3.6 mmHg (diastolic), thereby satisfying the stringent grade A of British Hypertension Society standard. This hydrogen-bond optimization strategy provides an avenue for piezoionic amplification and demonstrates the practicality of high-performance ionogels for wearable health-monitoring devices, thereby advancing the development of next-generation wearable biomedical systems.

## INTRODUCTION

Self-powered sensors capable of directly converting mechanical energy into electrical signals are increasingly recognized as a critical enabler toward the next generation of intelligent bio-integrated systems (*1*–*4*). In many biomedical scenarios, such sensors can enable zero-bias transduction at the sensing interface, reducing reliance on continuously powered sensing front ends and mitigating concerns associated with frequent battery replacement/recharging and potential leakage in long-term wearable use. Beyond signal transduction, wearable bio-integrated sensors must maintain skin-conformal contact and mechanical robustness under large deformation and repeated loading associated with daily motion. The conversion of mechanical energy to electrical power has typically been achieved through the piezoelectric effect (*5*) and the triboelectric effect (*6*). However, traditional piezoelectric materials are typically realized using brittle and lead-based ceramics, which respectively does not fit the soft nature of biological tissues, and are potentially toxic, thus limiting their suitability for bio-integrated systems (*7*, *8*). Furthermore, although triboelectric devices are known to be efficient energy converters, they tend not to be suitable for sensing as they are not sensitive to small stimuli (*9*, *10*) and they do not operate effectively at low frequencies (*11*), which are typical characteristics of biophysiological signals.

In recent years, the piezoionic effect has emerged as a promising self-powered sensing and energy-harvesting paradigm (*12*–*16*). Piezoionic conductors (e.g., hydrogels and ionogels) generate electrical outputs via pressure-induced separation of cations and anions (*17*–*19*). Piezoionics has been shown to be the only alternative that concurrently satisfies the requirements of self-power, accurate sensing, and realizable in soft materials (*13*, *20*, *21*). Madden and colleagues (*20*) first demonstrated the application of the piezoionic effect in a neural interface and further provided a systematic description of the energy and power transfer, relating these metrics to the piezoionic coefficient. Although such studies highlight the potential of piezoionic transduction, the electrical output of many piezoionic materials is limited, hindering practical applications. Our earlier work elucidated the relationships between piezoionic voltage generation and extrinsic operating parameters, such as stimulus strength and device architecture (*22*). Several works have introduced explicit piezoionic amplification. Here, “piezoionic amplification” refers to an intrinsic enhancement to the materials that result in an increased piezoionic response, rather than from electronic signal amplification (external to the material). Wu and colleagues (*21*) developed a device on the basis of the poly(vinylidene fluoride-*co*-hexafluoropropylene)/ionic liquid (IL) material. Their technique promoted charge separation via stress concentration by incorporating the rigid fillers within the polymer matrix. This modulus mismatch induces stress concentration at the filler-matrix interfaces under pressure. Stress concentration generates and propagates microcracks at the interface. (*23*, *24*) The accumulation of localized damage can degrade the stability of the devices (e.g., baseline drift). In our previous work, to preserve mechanical durability, we instead used a chemical approach that can preserve the homogeneity of the material. Crown ethers were used as a means of selective ion mobility enhancement, achieving a 30-fold piezoionic amplification in poly(vinyl alcohol) (PVA) hydrogels (*25*). However, the use of crown ethers does not improve mechanical properties. Therefore, there is yet a strategy that can provide piezoionic amplification and enhance mechanical properties simultaneously and without notable trade-offs.

PVA ionogels have attracted great attention recently due to their highly tunable chemical, mechanical, and ionic properties (*26*–*29*). The hydroxyl-rich backbone of PVA provides hydrogen bonding sites for ions, thereby opening the possibility to selectively immobilize one polarity of ions, e.g., the counterion, while allowing the primary ion to move freely, thus enhancing the cation-anion mobility difference to amplify the piezoionic output. However, conventional PVA gels, where designs have focused primarily on mechanical performance, typically rely on lengthy freeze-thaw processes (*30*) or small-molecule cross-linking (*31*). These processes lead to dense intra- and interchain hydrogen bonds (H-bonds; i.e., PVA-PVA), rather than H-bonds that are available to interact with ions. As a result, these processes render the PVA material highly rigid and poor in ionic conductivity, demanding previously unknown methods specifically for the requirements of piezoionic materials and devices (*28*, *32*).

**Fig. 1.**
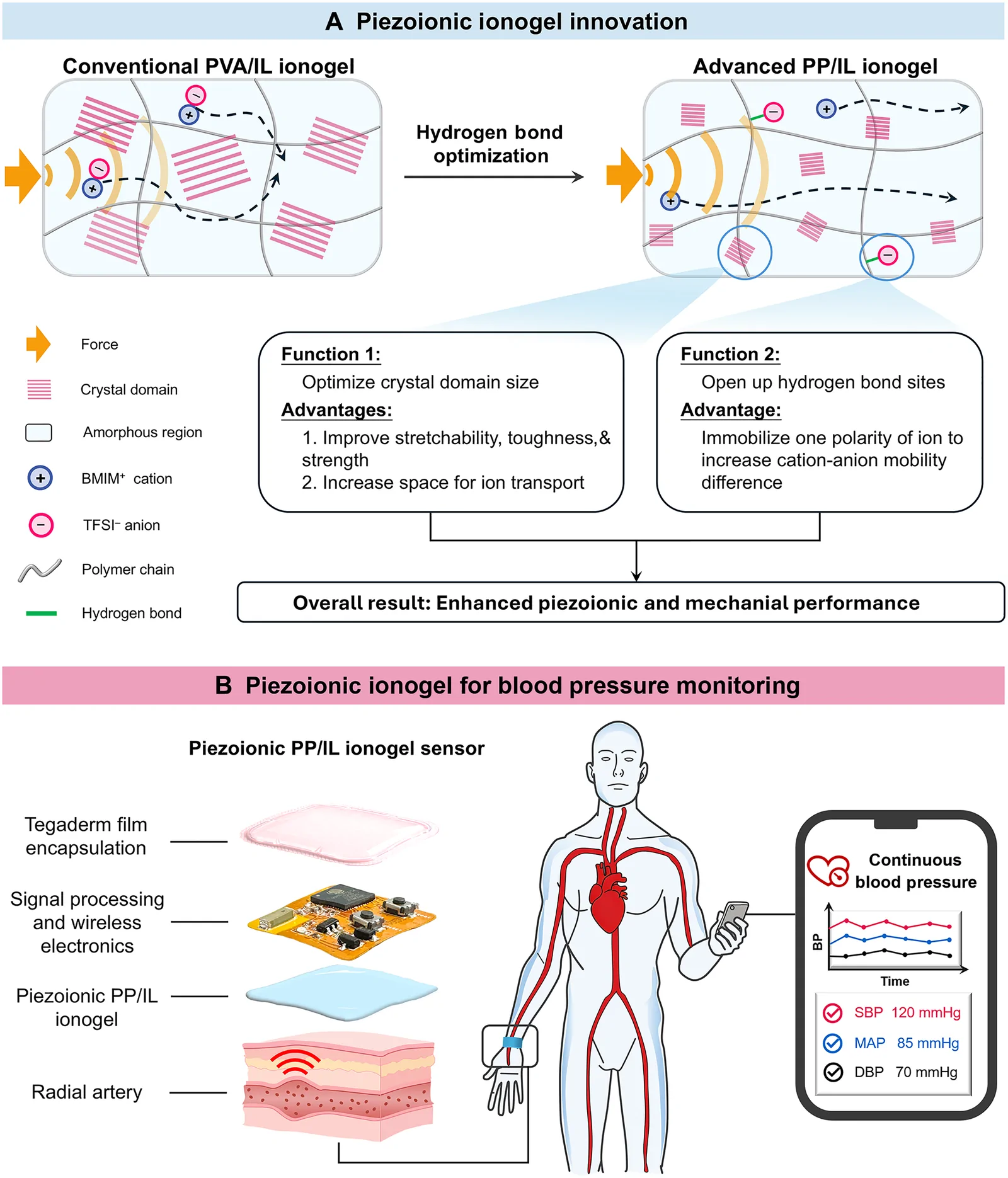
Design principle and application demonstration of piezoionic ionogel. (**A**) Materials innovation and associated advancements. Compared to conventional PVA/IL ionogel, the advanced PVA-PU/IL (PP/IL) ionogel achieves two major functions, namely, optimizing crystal size and exposing hydrogen bonding sites, thereby improving both mechanical and piezoionic performance. (**B**) Skin-wearable sensor structure and application demonstration in continuous blood pressure (BP) diagnostics. Exploded schematic of the wearable sensing system, consisting of an encapsulation layer, the piezoionic ionogel sensing layer, and integrated signal-processing and wireless electronics. When attached above a superficial artery (e.g., wrist), periodic vascular expansion induces dynamic deformation of the ionogel and generates a piezoionic output corresponding to the pulse waveform, enabling continuous BP estimation, including systolic BP (SBP), diastolic BP (DBP), and mean arterial pressure (MAP).

In this work, we present a hydrogen bond optimization strategy based on the copolymerization of PVA and polyurea (PU), aimed at significantly improving piezoionic amplification and mechanical properties. To the best of our knowledge, this is the first report of a piezoionic copolymer and the engineering of its hydrogen bond network for explicit piezoionic amplification and enhanced mechanical properties. As shown in Fig. 1A, the copolymerization of PVA and PU reconfigures the H-bond network and, thus, achieves two main objectives: (i) optimizing the crystallinity to enhance ion transport and mechanical properties; and (ii) selectively immobilizing the counterion, thus amplifying the cation-anion separation. The copolymer ionogel and associated self-powered pressure sensor prototypes were fabricated to achieve a piezoionic output of 20 mV, which is a 10-fold improvement after H-bond optimization. It also exhibits excellent stretchability (1510%), toughness (23.1 MJ m^−3^), and durability. For practical application, a self-powered epidermal pressure sensor was fabricated and integrated with signal processing circuits and machine learning algorithms into a cuffless, noninvasive continuous blood pressure (BP)–monitoring system, achieving an accuracy that meets the grade A of British Hypertension Society (BHS) standard. Figure 1B depicts the system architecture and application scenario. The proposed strategy represents, to the best of our knowledge, the first demonstration that brings piezoionics out of the scientific realm and into the world of practical wearable intelligent biomedical devices.

## RESULTS

### Synthesis and structural characterization of piezoionic ionogels

**Fig. 2.**
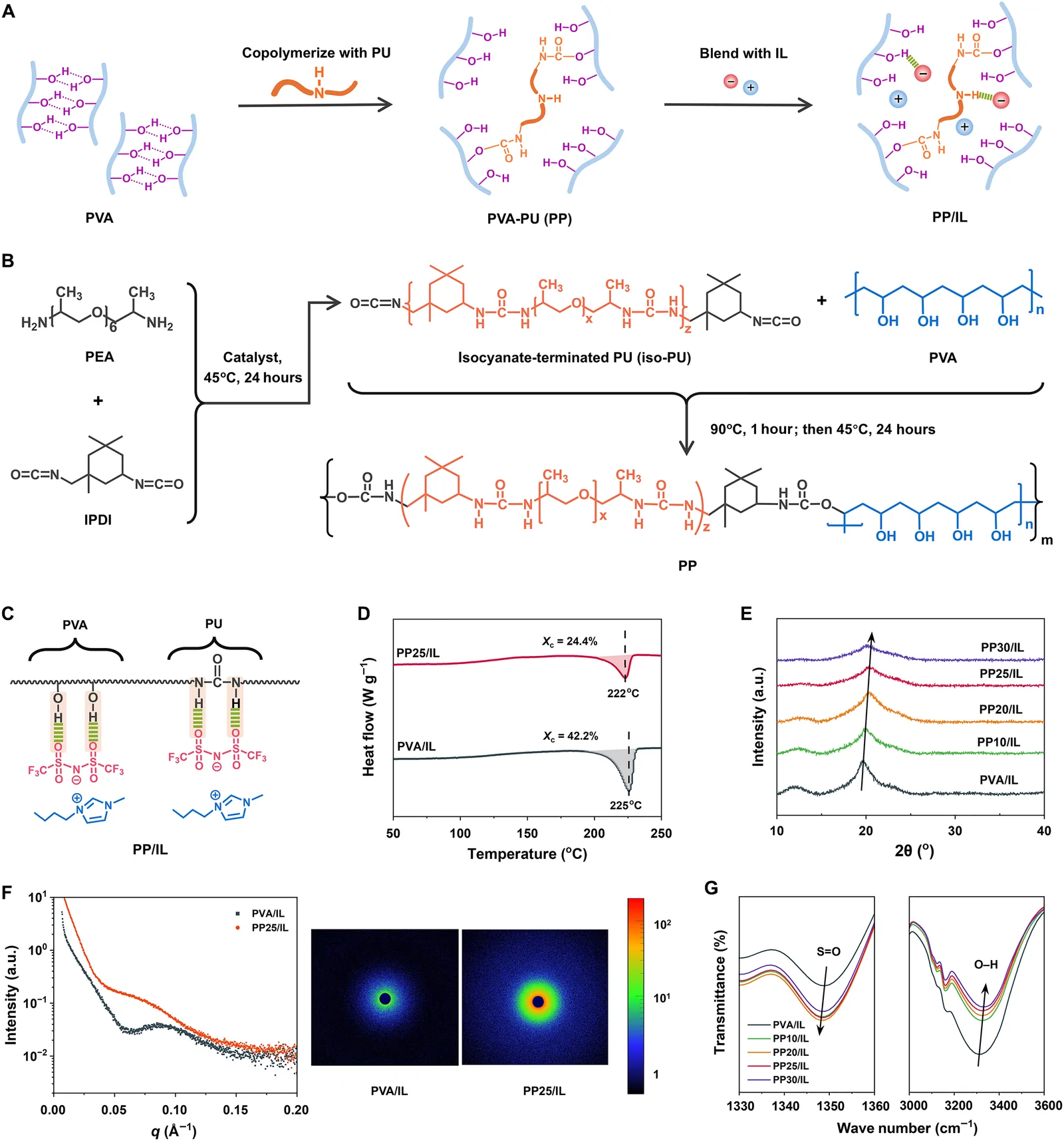
Synthesis and structural characterization of piezoionic ionogels. (**A**) Design rationale and working mechanism: PVA is copolymerized with PU segments to form a PP copolymer, which reconstructs the hydrogen bond network. After introducing the IL, the optimized crystallinity and increased hydrogen bonding sites promote stronger polymer-anion interaction, which enlarges diffusion difference between cations and anions, thereby amplifying the piezoionic response. Synthesis process of (**B**) PP copolymer. (**C**) Schematic of ion-polymer interactions in PP/IL, highlighting multivalent hydrogen-bonding interactions between ions and ─OH/─NH groups. (**D**) Differential scanning calorimetry (DSC) curves of ionogels to assess crystallinity changes induced by PU incorporation, (**E**) x-ray diffraction (XRD) patterns of ionogels with increasing PU content, showing the evolution of average crystallite size. (**F**) Small-angle x-ray scattering (SAXS) spectra and corresponding 2D patterns, revealing average interdomain distance and spatial distribution of crystal domains in PP/IL relative to PVA/IL. (**G**) Fourier transform infrared (FTIR) spectra displaying peak shifts, evidencing altered hydrogen bonding and polymer-ion interactions in PP/IL ionogels.

Hydrogen bond optimization of PVA enhances mechanical properties and amplifies the piezoionic response simultaneously. This chemical reconstruction fundamentally transforms the polymer into a functional matrix that actively drives ion transport to amplify the piezoionic response. As shown in Fig. 2A, through the copolymerization of PVA and PU to obtain the desirable molecular structure, the strategy achieves two essential goals: (i) tuning of crystallinity, thereby improving toughness, strength, and stretchability, as well as enhancing ion transport; and (ii) liberating H-bonds that are otherwise wrapped up between PVA chains to be free to act as active sites for selectively immobilizing anions, thereby amplifying the cation-anion mobility difference.

To realize the above strategy, a custom isocyanate-terminated PU (iso-PU) prepolymer was synthesized by reacting isophorone diisocyanate (IPDI) with a low–molecular-weight polyetheramine (PEA) and then covalently reacted with PVA hydroxyls to form urethane linkages, yielding a PVA-PU copolymer (PP). Instead of a physical blend, copolymerization enables the chemical tailoring of molecular structure (Fig. 2B and fig. S1). This covalent incorporation optimizes dense PVA-PVA hydrogen bonding and lowers crystallinity, thereby facilitating ion transport and liberating more ─OH sites that can participate in selective anion immobilization. To obtain a marked disparity in cation and anion diffusivities, we selected 1-butyl-3-methylimidazolium bis(trifluoromethylsulfonyl)imide ([BMIM][TFSI]) as the IL to prepare the PP/IL ionogel, as shown in Fig. 2C. The TFSI^−^ anion, with multiple sulfonyl oxygen acceptors and a highly delocalized charge, acts as a hydrogen-bond acceptor to PP donor sites (principally ─OH from PVA and ─NH from PU), enhancing the interaction between the anion and the polymer chains, whereas BMIM^+^ remains comparatively mobile within the PP matrix. To realize the above strategy, we investigated a series of PP/IL ionogels with varying PU contents (PP10/IL, PP20/IL, PP25/IL, and PP30/IL). The synthesis procedures and compositions of the PP/IL ionogels are detailed in Materials and Methods.

The crystallization behavior of PP/IL ionogels was investigated using differential scanning calorimetry (DSC), x-ray diffraction (XRD), and small-angle X-ray scattering (SAXS). Specifically, the degree of crystallinity (*Χ*_c_) was calculated from DSC results: *Χ*_c_ (%) = [Δ*H*_m_/(Δ*H*_m₀_ × *w*_PVA_)] × 100%, where Δ*H*_m_ is the melting enthalpy obtained by integrating the DSC melting endotherm, Δ*H*_m₀_ is the melting enthalpy of 100% crystalline PVA, and *w*_PVA_ is the mass fraction of PVA in the ionogel. As shown in Fig. 2D, the PVA/IL ionogel, which contains PVA as the sole polymer component, exhibits a crystallinity of 42.2%. Upon incorporation of PU, the crystallinity of the PP25/IL ionogel decreases to 24.4%, which can be attributed to the disruption of crystalline domains and the increased amorphous regions. The melting peak (*T*_m_) of PVA/IL appears at 225°C, whereas that of PP25/IL is at a lower value of 222°C, which can be attributed to the decrease in crystallinity.

In XRD analysis, for the PVA/IL ionogel, a pronounced diffraction peak appears at 2θ = 19.7°, corresponding to the (101) reflection plane characteristic of semicrystalline PVA, as shown in Fig. 2E. Upon copolymerization with PU, the intensity of this peak gradually decreases, indicating a reduction in crystallinity, consistent with DSC data. The average crystallite size (*D*) was calculated using the Scherrer equation, *D* = *k* λ/(β cos θ) (*33*), where *k* is the shape factor (0.9), λ is the x-ray wavelength, β is the full width at half maximum, and θ is the Bragg angle. The *D* decreases significantly from 2.46 nm for PVA/IL to 1.73 nm for PP25/IL upon PU copolymerization. As shown in Fig. 2F, SAXS analysis further reveals broad peaks at *q* = 0.085 and 0.068 for PVA/IL and PP25/IL, respectively, showing crystalline domains in both systems, albeit with different sizes. The average interdomain distance (*L*), calculated from the equation (*L* = 2π/*q*) (*34*), is slightly larger for PP25/IL (9.23 nm) than for PVA/IL (7.4 nm). Additionally, it can be observed from the two-dimensional (2D) SAXS pattern that the broader and more intense scattering in the small-angle region for PP25/IL suggests a more uniform spatial distribution of crystal domains.

Fourier transform infrared (FTIR) spectroscopy, as shown in Fig. 2G, was used to characterize the molecular interactions within the PVA/IL and PP/IL ionogels. The O─H stretching vibration of the PVA/IL ionogel is observed at 3314 cm^−1^, whereas that of the PP/IL ionogel is at 3333 cm^−1^, i.e., blue-shifted. This indicates that the copolymerization with PU led to the dissociation or weakening of H-bonds formed between the ─OH groups in PVA. Simultaneously, the S═O vibration of TFSI^−^ is red-shifted from 1350 to 1342 cm^−1^, suggesting increased involvement of TFSI^−^ in H-bond formation, which immobilizes it onto the polymer backbone. Thermogravimetric analysis (TGA) further confirmed the good thermal stability of the PP/IL ionogel, with a 5% weight-loss temperature (*T*_d,5%_) of 262°C (fig. S2).

In short, copolymerization with PU reconfigures the hydrogen-bond network, which achieves two main functions. First, it reduces the crystalline domain size, thus increasing the amorphous fraction, thereby improving ion-transport efficiency. Second, the enlarged amorphous regions provide more accessible ─OH sites. The TFSI^−^ counterion can provide hydrogen-bond acceptor sites owing to its delocalized negative charge and multiple sulfonyl oxygen atoms bearing lone-pair electron density, which can form hydrogen bonds with ─OH donor sites in PVA, thereby strengthening anion-polymer interactions. This hydrogen bonding with the TFSI^−^ counterion helps immobilize them onto the polymer backbone, thereby increasing the diffusion difference between cations and anions under applied pressure, which forms the basis for enhanced piezoionic performance. This result is subsequently supported by density functional theory (DFT) and molecular dynamics (MD) simulations.

### Mechanical properties and piezoionic amplification of PP/IL ionogel

**Fig. 3.**
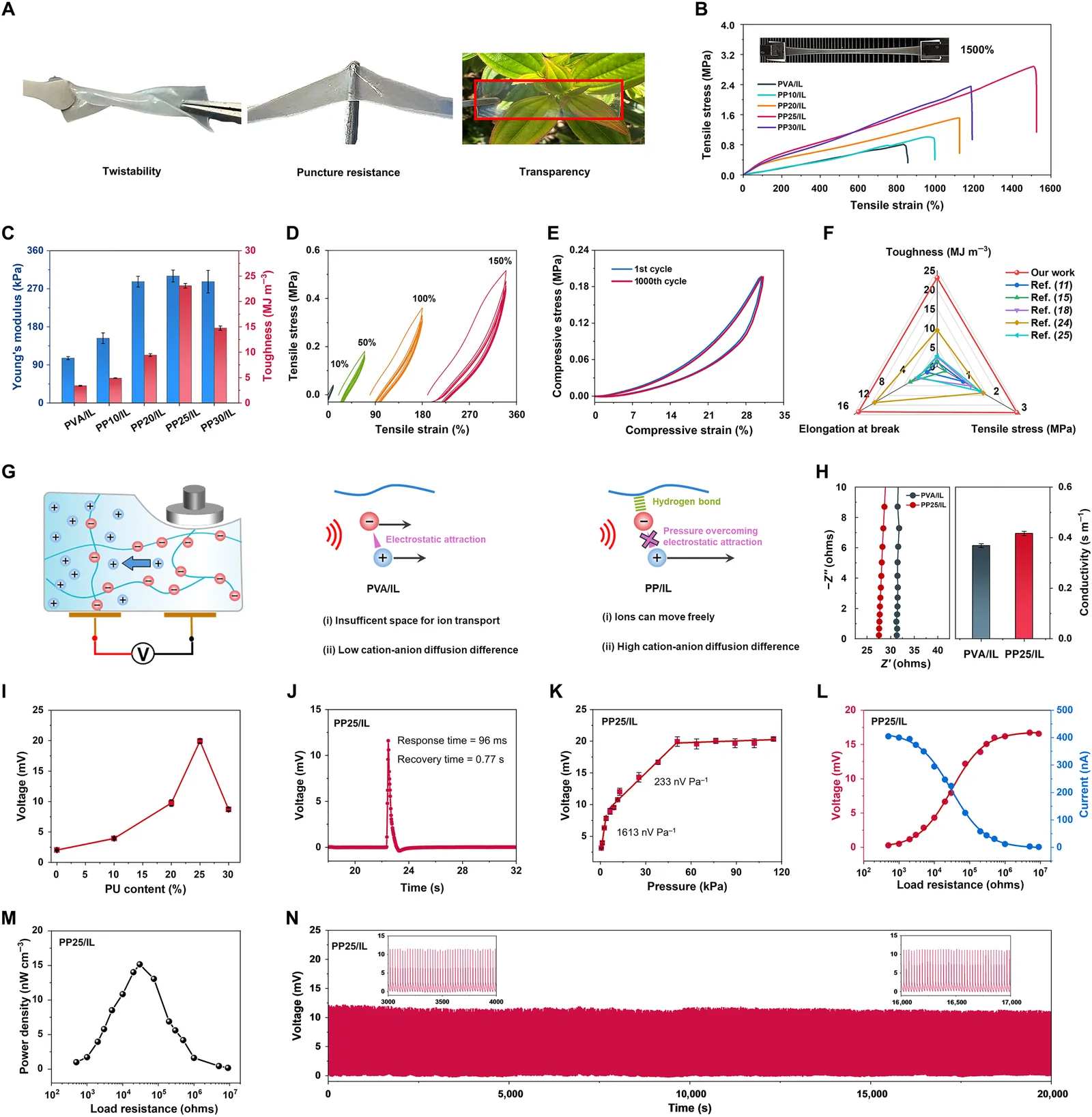
Mechanical properties and piezoionic amplification of PP/IL ionogel. (**A**) Photographs of the PP25/IL ionogel, a PP/IL ionogel prepared with a PVA:PU mass ratio of 75:25 (5 cm in length, 2 cm in width, and 0.5 mm in thickness). A remarkable twistability was exhibited under manual deformation. In the puncture resistance test with a metallic needle, no through-hole was observed. The ionogel, highlighted using a red box, does not significantly obstruct the background, showing good transparency. (**B**) Stress-strain curve of PP/IL ionogels with different PU contents, together with optical images of PP25/IL ionogels at 1500% elongation. (**C**) Young’s modulus and toughness of PP/IL ionogels with different PU contents extracted from the tensile curves in (B) (data are means ± SD, *n* = 3). (**D**) Cyclic tensile loading-unloading behavior of PP25/IL at increasing strains. (**E**) Compressive cycling performance of PP25/IL at the 1st and 1000th cycles. (**F**) Comparison of the PP25/IL ionogel with previously reported piezoionic materials in terms of tensile stress, elongation at break, and toughness. (**G**) Schematic diagram of piezoionic signal generation under a pressure stimulus: Compared with conventional PVA/IL, the PP/IL ionogel allows the freer motion of cations and promotes a high cation-anion diffusion difference via selectively anion immobilization. The blue arrow indicates the direction of cation migration. (**H**) Electrochemical impedance spectra and derived ionic conductivity of PVA/IL and PP25/IL ionogels (data are means ± SD, *n* = 3). (**I**) Voltage response of PVA/IL and PP/IL ionogels with different PU contents at 50 kPa, revealing an optimal composition for piezoionic output (data are means ± SD, *n* = 5). (**J**) Voltage response of PP25/IL ionogel at 13 kPa, with the corresponding response and recovery times. (**K**) Pressure-dependent voltage output of PP25/IL ionogel over a broad pressure range (data are means ± SD, *n* = 4). Load-dependent output characteristics of PP25/IL at 50 kPa: (**L**) voltage and current, and (**M**) power density as a function of external load resistance. (**N**) Cyclic stability of PP25/IL ionogel under repeated pressure loading (13 kPa), demonstrating stable and repeatable piezoionic output.

As shown in Fig. 3A, the PP/IL ionogels are highly flexible, are easily twisted, and exhibit excellent puncture resistance, fulfilling structural requirements for wearable sensors. The PP/IL ionogel also exhibits high transmittance (fig. S3). Tensile tests were conducted to characterize mechanical properties, including tensile strength, Young’s modulus, toughness, and elongation at break, as shown in Fig. 3 (B and C). With increasing PU content, tensile strength, toughness, and elongation at break initially rise to an optimal point then decline. The PP25/IL ionogel can be stretched up to 15 times its original length. The mechanism of improved stretchability is illustrated in fig. S4. The copolymerization-driven redistribution from densely aggregated PVA-PVA hydrogen-bonded crystalline domains toward a network of stable and dynamic PVA-PU interactions that can dissociate and reorganize under stress, allowing the chains to stretch in a certain direction without immediate fracture, thus imparting excellent strength, stretchability, and toughness. With increasing PU content, Young’s modulus increases for the following reasons. The ─NH groups from PU that form extensive strong hydrogen-bonding interactions with PVA and with the TFSI^−^ anions effectively increase the density of physical cross-links and in the small-strain regime. In addition, the rigid isophorone rings from PU can restrict polymer segmental motion (see fig. S5). Therefore, the enhanced polymer-polymer and ion-polymer interactions, combined with rigid ring, increase network connectivity and resistance to deformation, resulting in an increased Young’s modulus. The PP25/IL ionogel achieves optimal mechanical properties, with a tensile strength of 3.0 MPa, elongation at break of 1510%, Young’s modulus of 300 kPa, and toughness of 23.1 MJ m^−3^. Excessive PU content (PP30/IL) leads to notable phase separation, disrupting load transfer and creating stress concentration sites, thus degrading mechanical properties.

As shown in Fig. 3D, fatigue durability tests indicate that PP25/IL ionogels undergo five loading-unloading cycles at different strains, with the first cycle showing a pronounced hysteresis loop due to energy dissipation from reversible H-bond rupture, which is typical. The overlapping curves from the second to the fifth cycles indicate rapid reformation of dynamic bonds and minimal damage accumulation, confirming excellent fatigue resistance. The hysteresis area increases as strain rises from 100 to 150%, reflecting greater energy dissipation at higher deformations. The compressive stress-strain curves of the PVA/IL and PP/IL ionogels are shown in fig. S6. All ionogels do not fracture at the compressive strain of 80%.

Cyclic compression tests in Fig. 3E show negligible changes in both the baseline and response amplitude after 1000 cycles, further confirming the structural stability of the PP25/IL ionogel. Compared with previously reported piezoionic materials as shown in Fig. 3F and table S1, PP25/IL ionogel achieves high Young’s modulus, toughness, and elongation at break, achieving excellent mechanical properties that form a solid foundation for excellent electrical response, as detailed subsequently (*15*, *21*, *25*, *35*, *36*). Furthermore, the PP25/IL ionogel was evaluated against gels with high mechanical properties, as shown in table S2. The ionogel combines high stretchability with a low Young’s modulus (*29*, *33*, *37*). This mechanical profile is particularly beneficial for epidermal pressure sensing (e.g., wrist pulse monitoring for cuffless BP analysis), where the device experiences long-term cyclic, small-amplitude compression and must resist handling-induced deformation during daily wear. In this context, sufficient modulus and high toughness help preserve sensor geometry and interfacial integrity, thereby reducing artifacts and baseline drift, while the large stretchability ensures conformal contact and user comfort.

The systematic engineering of piezoionic amplification through counterion (anion) immobilization onto the polymer backbone is illustrated in Fig. 3G and fig. S7. The enhancement of the piezoionic properties of the PP/IL ionogel through H-bond optimization has two synergistic effects: (i) promotes the formation of amorphous regions, which is the space for effective ion transport; and (ii) liberates the hydroxyl groups to act as preferential interaction sites with the TFSI^−^ anions. Such preferential binding attenuates TFSI^−^ mobility while preserving relatively high BMIM^+^ mobility, increasing the cation-anion mobility difference, thus amplifying the piezoionic effect. The higher mobility of BMIM^+^ in the piezoionic ionogel was identified by selecting the electrode to which pressure was applied, as shown in movie S1.

As shown in Fig. 3H, electrochemical impedance spectroscopy (EIS) measurements reveal that PP25/IL ionogel exhibits an ionic conductivity of 0.42 S m^−1^, an improvement from the 0.37 S m^−1^ of PVA/IL ionogel. It should be noted that ionic conductivity is not a sole contributor of the piezoionic voltage, which is governed primarily by the diffusion difference between cations and anions; the conductivity data here are provided to confirm that hydrogen-bond optimization promotes ion transport. To determine the optimal composition for piezoionic performance, the piezoionic responses of PP/IL ionogels are further evaluated. As shown in Fig. 3I and fig. S8, a 25% PU content produces the highest piezoionic output. The achieved peak piezoionic output, at 50 kPa, delivers a voltage of 20 mV, markedly a one order of magnitude improvement from the conventional pristine PVA ionogels of 2 mV. When the PU content is excessively high at 30 wt %, the sample exhibits degradation in mechanical properties, resulting in a drop in piezoionic performance. Figure 3J shows that the voltage response of the PP25/IL ionogel at 13 kPa is 12 mV. From the voltage-time curve, the response and recovery times of the PP25/IL ionogel are calculated to be 96 ms and 0.77 s. Figure 3K presents the voltage variation of the PP25/IL ionogel under different pressures. In the low-pressure regime, the ionogel network undergoes slight compaction. The local ion-transport environment is still highly sensitive to pressure. Small increases in pressure can therefore produce large changes in the relative mobility of BMIM^+^ and TFSI^−^. The voltage increases rapidly with pressure, giving a relatively steep initial slope. A high piezoionic coefficient of 1613 nV Pa^−1^ is obtained in this region. In the intermediate-pressure regime, the polymer network becomes more densified and the hydrogen-bond interactions responsible for TFSI^−^ immobilization become more stable. The pressure-induced enhancement of ion transport asymmetry slows down, causing the response curve to maintain its linearity but at a reduced gradient. At high pressure, the available pathways for ion transport become strongly limited, and the pressure-induced enhancement in differential ion migration tends toward saturation. Consequently, the voltage increment per unit pressure decreases markedly, giving rise to the observed plateau. We further evaluated the sensor under elevated compressive loads to assess high load tolerance. The PP25/IL device remains functional up to 500 kPa and continues to produce a clear transient piezoionic response of 21 mV (fig. S9), with a tendency toward response saturation at higher pressures.

Power density analysis of the PP25/IL ionogel is explored as shown in Fig. 3 (L and M), demonstrating self-powered capability. The output voltage steadily increases with the external load resistance, while current decreases. The peak power density of 15.2 nW cm^−3^ is achieved, highlighting its potential not only as a self-powered sensor but also as an energy harvester. These dual-sensing and energy-harvesting capabilities are especially synergetic for environmental and biomedical applications. For cycling stability, as shown in Fig. 3N, the PP25/IL ionogel maintains nearly constant output after 20,000 s of compression cycles, underscoring their structural robustness. The results suggest that the sensor exhibits minimal hysteresis. Consequently, no additional history compensation is required for routine tests (such as pulse/BP monitoring) beyond letting the sensor settle for several seconds after device mounting before data recording. As shown in table S3, compared with previously reported piezoionic materials (*20*, *21*, *25*, *38*–*40*), the PP25/IL ionogel exhibits a significantly higher piezoionic coefficient, an excellent pressure response range, and a relatively short response time, which are advantageous for biosensing applications.

### Mechanism of H-bond optimized piezoionic amplification

**Fig. 4.**
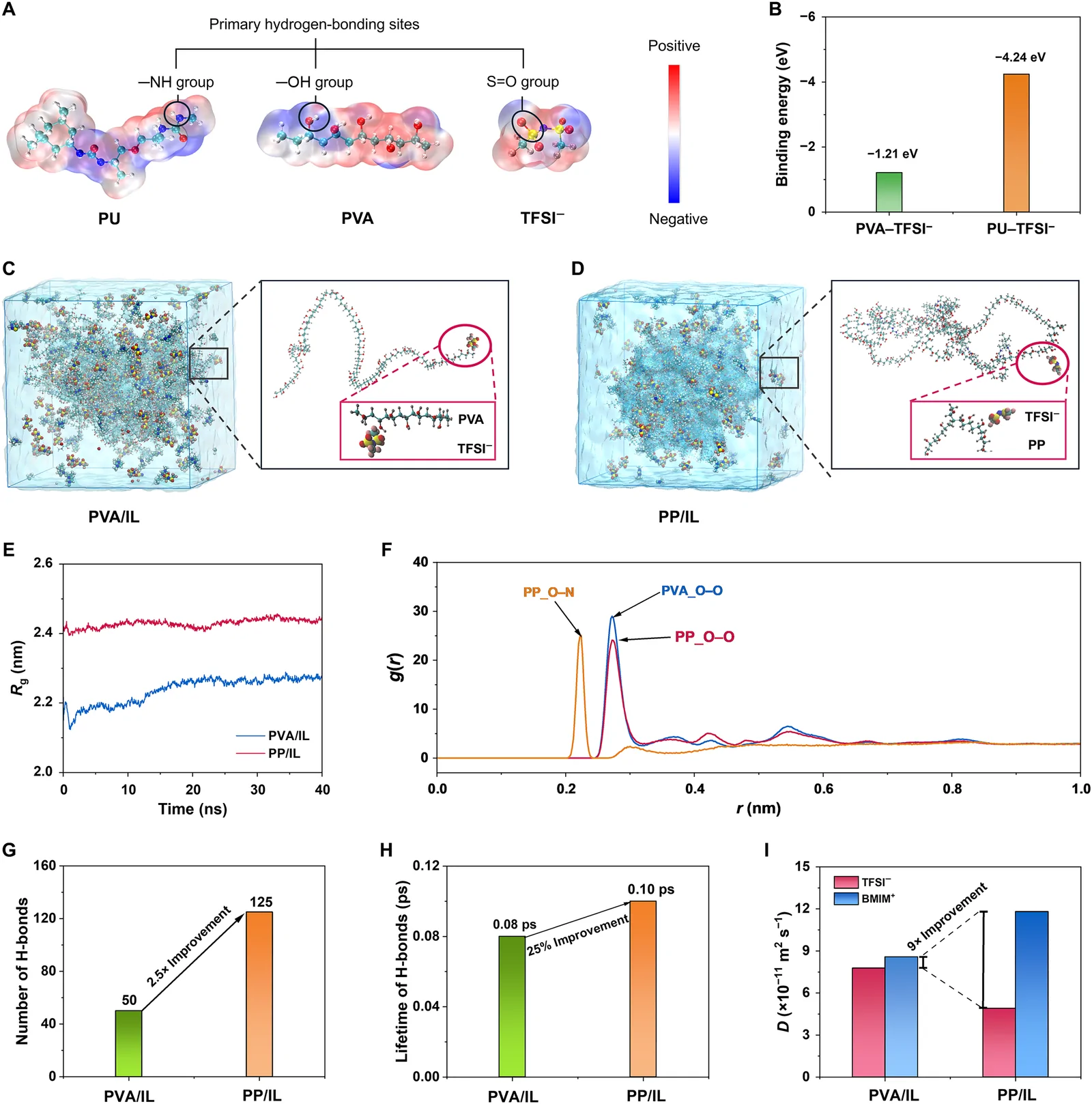
Hydrogen-bond regulation and ion transport in PP/IL ionogels revealed by simulations. (**A**) Electrostatic potential (ESP) of the PVA unit, PU unit, and TFSI^−^ anion, identifying ─OH, ─NH, and S═O groups as primary hydrogen-bonding donor/acceptor sites. (**B**) Calculated binding energies for ion-polymer unit, showing markedly stronger PU-TFSI^−^ interaction (−4.24 eV) than PVA-TFSI^−^ (−1.21 eV). Snapshots from molecular dynamics (MD) simulations of (**C**) PVA/IL ionogel and (**D**) PP/IL ionogel under an applied pressure of 15 kPa, illustrating ion-polymer interaction states. (**E**) Time evolution of the radius of gyration (*R*_g_), indicating a more open chain conformation in PP/IL (*R*_g_ ≈ 2.40 nm) compared with PVA/IL (*R*_g_ ≈ 2.25 nm). (**F**) Radial distribution function (RDF) curves of PVA/IL and PP/IL ionogels, revealing reconfigured short-range order upon PU copolymerization. (**G**) Number of hydrogen bonds extracted from MD, showing a pronounced increase in PP/IL compared with PVA/IL. (**H**) Hydrogen-bond lifetime in PVA/IL and PP/IL, revealing more persistent hydrogen-bonding interactions in PP/IL. (**I**) Diffusion coefficients of ionic species (TFSI^−^ and BMIM^+^) in PVA/IL and PP/IL, demonstrating altered diffusion difference upon PU incorporation and supporting the experimentally observed enhancement of piezoionic performance.

To elucidate the molecular-level interactions responsible for the enhanced piezoionic output, we performed DFT calculations to analyze the electrostatic potential (ESP) distributions of representative structural motifs (PVA unit and PU unit) from PP copolymer and the TFSI^−^ anion, as shown in Fig. 4A. The ─OH in PVA unit and the ─NH in PU unit exhibit pronounced polarity, with electronegative O or N atoms bearing negative ESPs and the corresponding H atoms bearing positive potentials. Similarly, the S═O in TFSI^−^ are strongly polarized, with oxygen atoms having pronounced negative ESPs, identifying these as preferred interaction sites with H atoms from PVA unit and PU unit. To further quantify the interaction strength, the binding energies of the PU unit with TFSI^−^ (PU-TFSI^−^) and the PVA unit with TFSI^−^ (PVA-TFSI^−^) were calculated to be −4.24 and −1.21 eV, respectively, as shown in Fig. 4B and fig. S10. Results confirm that both PVA unit and PU unit exhibit hydrogen-bonding interaction sites with TFSI^−^, whereas the significantly stronger PU-TFSI^−^ binding indicates greater stability compared with PVA-TFSI^−^. This comparative trend supports the enhanced immobilization of TFSI^−^ in PP/IL through strengthened hydrogen-bonding interactions. The DFT results are used as mechanistic support to corroborate the experimental FTIR and the MD simulations.

MD simulations were conducted to investigate ion transport in PVA/IL and PP/IL ionogels under an applied pressure of 15 kPa, as shown in Fig. 4 (C and D). The structural evolution of the polymer network is characterized through the radius of gyration (*R*_g_) and radial distribution function (RDF), which respectively provide quantitative insights into polymer chain conformation and local atomic arrangement. Figure 4E demonstrates that the PVA/IL ionogel has polymer chains with a small *R*_g_ of 2.25 nm, indicating a compact conformation with restricted chain mobility. Figure 4F shows that the O─O RDF, where both oxygen atoms are from the PVA hydroxyl groups, exhibits a sharp, intense peak at 0.272 nm with *g*(*r*) = 28.9, indicating pronounced short-range hydrogen-bonded ordering among hydroxyl groups, consistent with the presence of crystalline domains observed in the XRD data. Such dense H-bonded crystalline domains limit the accessibility of hydroxyl groups for coordinating with ions. For the model used in Fig. 4 (G and H) and figs. S11 and S12, only 50 polymer-TFSI^−^ hydrogen bonds are formed, with an average lifetime of 0.08 ps. This insufficient anion coordination leads to poor TFSI^−^ immobilization, permitting the anions and cations to transport as pairs, thus producing a weak piezoionic effect. In PVA/IL, the diffusion coefficients of BMIM^+^ (8.57 × 10^−11^ m^2^ s^−1^) and TFSI^−^ (7.78 × 10^−11^ m^2^ s^−1^) yield a diffusion difference (*D*_d_ = *D*_BMIM_^+^ − *D*_TFSI_^−^) of only 0.79 × 10^−11^ m^2^ s^−1^, as shown in Fig. 4I and fig. S13.

Upon copolymerization with PU chains, a notable reconfiguration of the H-bond network was observed. A decrease in the O─O RDF peak intensity at 0.272 nm from *g*(*r*) = 28.9 to *g*(*r*) = 24.1, the emergence of an O─N peak at 0.22 nm with *g*(*r*) = 25.0, and an increase in *R*_g_ from 2.25 to 2.40 nm together indicate that copolymerization with PU reduces short-range hydroxyl ordering and introduces N─H─O hydrogen bonds. This increased short-range structural heterogeneity, which is highly desirable. As indicated by the *R*_g_ and RDF results, partial chain loosening and reduced hydroxyl ordering increase the availability of hydroxyls as interaction sites for TFSI^−^, significantly increasing the polymer-TFSI^−^ H-bonds to 125 in our model, representing a 150% increase compared to PVA/IL. Additionally, the H-bond lifetime also increases by 25% from 0.08 to 0.10 ps, as shown in Fig. 4H, further supporting a strengthening in TFSI^−^ immobilization. TFSI^−^ immobilization significantly reduces its diffusion coefficient to 4.91 × 10^−11^ m^2^ s^−1^. Conversely, the reduction of dense crystalline packing facilitates BMIM^+^ motion and increases its diffusion coefficient by 38% to 11.8 × 10^−11^ m^2^ s^−1^. As a result, the *D*_d_ increased sharply to 6.89 × 10^−11^ m^2^ s^−1^, which is a remarkable ninefold improvement.

PU copolymerization reduces the dense hydrogen-bonded crystalline packing of PVA and creates a more amorphous, less sterically hindered transport environment, which promotes BMIM^+^ diffusion. At the same time, the reconfigured hydrogen-bond network liberates ─OH groups from PVA and introduces ─NH groups from PU, both of which form hydrogen bond with TFSI^−^. These hydrogen-bond interactions selectively retard anion motion. As a result, *D*_BMIM_^+^ increases, whereas *D*_TFSI_^−^ decreases, leading to the enhanced piezoionic response. The increase in *D*_BMIM_^+^ is consistent with the increase in ionic conductivity from 0.37 to 0.42 S m^−1^, reflecting facilitated ion transport in the more amorphous polymer matrix. Meanwhile, the decrease in *D*_TFSI_^−^ stems from the immobilization of TFSI^−^ via hydrogen bonding with the polymer matrix, which offsets part of the conductivity gain. As a result, the conductivity increases moderately. We note that piezoionic output depends on the diffusion difference between the cation and anion, rather than the absolute distance the ions traveled. Even if the fraction of anions is immobilized on the polymer backbone, such engineered immobilization greatly separates the ion from the counterion, enabling a larger generated piezoionic voltage under pressure. In this regard, the enhanced piezoionic response is attributable to two synergistic effects: (i) immobilization of the TFSI^−^ anions and (ii) the free move of BMIM^+^ cations. This phenomenon can be described as streaming potential (see note S3). As an additional benefit of copolymerization with PU, the immobilizing H-bonds in PVA/IL are formed predominantly between ─OH of PVA and the S═O of TFSI^−^. However, in PP/IL, an additional type of H-bond is evident between TFSI^−^ and the ─NH groups of PU, augmenting immobilization, which further enhances the piezoionic effect.

### Piezoionic ionogel for BP monitoring

**Fig. 5.**
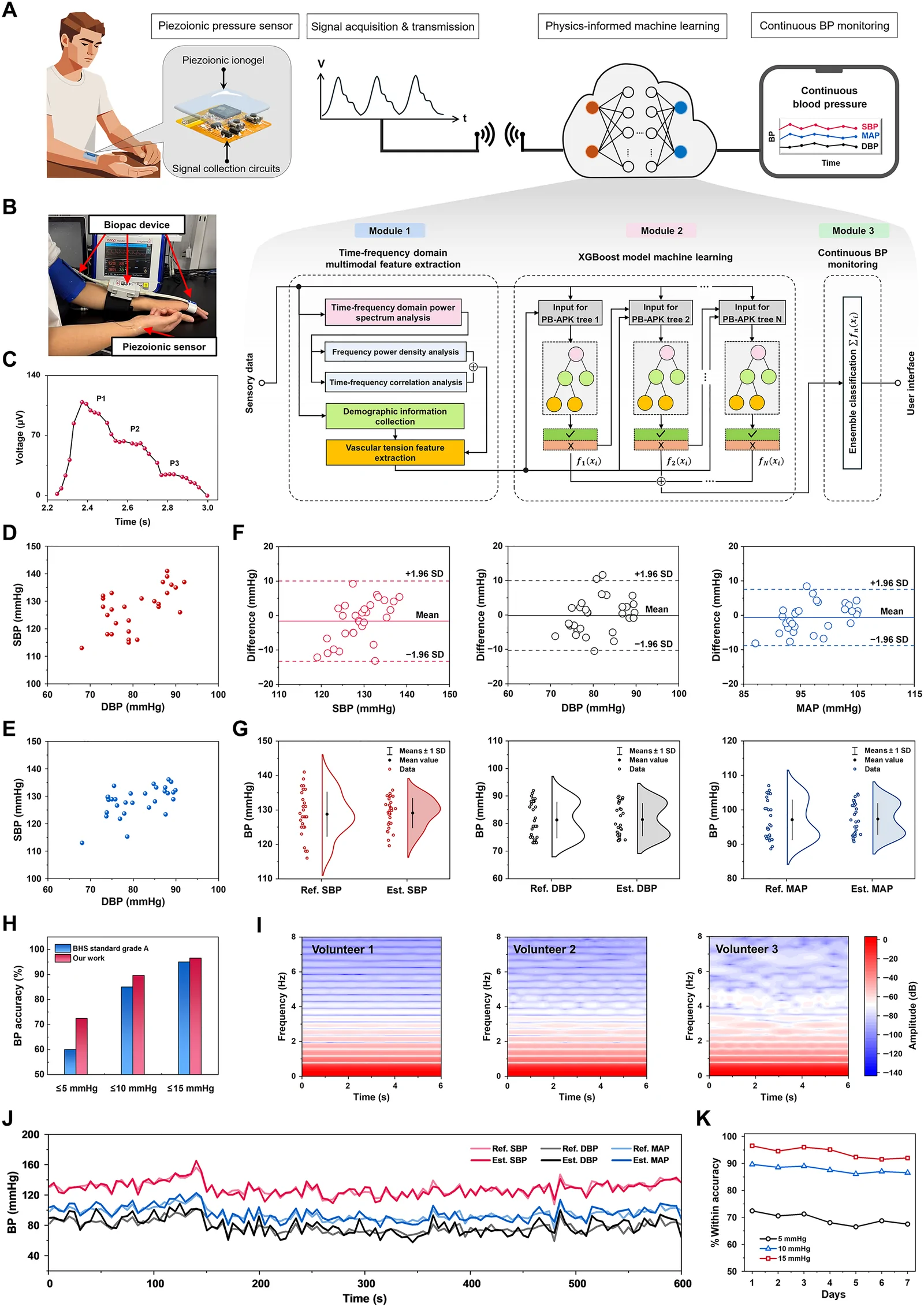
Cuffless, noninvasive, and continuous BP monitoring using a self-powered PP25/IL piezoionic ionogel sensor and physics-informed machine learning. (A) System concept: The wrist-mounted PP25/IL piezoionic sensor directly acquires pulse signals. A physics-informed learning model converts the pulse signal into SBP/DBP/MAP values for real-time display. The inset illustrates the three-module algorithm: module 1, time-frequency domain multimodal feature extraction combined with demographic information to derive a vascular-tension feature; module 2, XGBoost model machine learning to estimate SBP/DBP/MAP; and module 3, real-time output of SBP/DBP/MAP on a user terminal. The piezoionic sensor operates in a self-powered sensing mode. The current system uses external power for signal collection and machine learning. (B) Photograph of the calibration setup, with a commercial Biopac system providing reference measurements and the PP25/IL sensor simultaneously acquiring wrist pulse waveforms (C) Representative pulse waveform captured by the PP25/IL sensor, with obvious characteristic peaks. (D) Reference BP distribution of volunteers measured by Biopac and (E) corresponding BP estimates produced by the proposed system. (F) Bland-Altman plots for SBP, DBP, and MAP comparing estimated and reference values (solid lines, mean bias; dashed lines, 95% limits of agreement, ±1.96 SD). (G) Violin plots comparing the distributions of estimated and reference SBP/DBP/MAP. (H) Error analysis benchmarked against the BHS standard, reporting the percentage of estimates within ±5, ±10, and ±15 mmHg. (I) Time-frequency energy spectra from three representative volunteers, illustrating subject-dependent spectral characteristics. (J) Continuous BP monitoring for 600 s, showing stable cuffless tracking of SBP, DBP, and MAP compared to the corresponding reference values. (K) BP estimation accuracy over a seven-day period, demonstrating the long-term stability of the sensor.

Piezoionic ionogel sensors are ideally suited for wearable health monitoring due to their intrinsic properties, which include self-powered sensing, flexibility, skin-conformal interfaces, and high sensitivity to physiological signal frequencies. In addition to being used for sensor fabrication, the PP25/IL ionogel can also serve as a soft, skin-conformal electrode. When used as an electrode to record electrocardiogram (ECG) signals, it maintains reliable contact to deliver stable, high-fidelity ECG waveforms both before and after exercise, as shown in fig. S14 and note S4. As illustrated in fig. S15, the self-powered PP25/IL sensor consists of a PP25/IL ionogel and circular metal electrodes. Then, the self-powered PP25/IL sensor integrated with flexible electronic circuits and machine learning algorithms to construct a cuffless, noninvasive, and continuous BP-monitoring system. As shown in Fig. 5A and note S5, the PP25/IL sensor attached to the wrist acquires arterial pulse signals. The physics-informed machine learning algorithm uses collected pulse waveform data to estimate systolic BP (SBP)/diastolic BP (DBP)/mean arterial pressure (MAP) enabling continuous BP monitoring and real-time display on a terminal. Unlike conventional noninvasive pulse-monitoring techniques (*41*, *42*), including optical method (*43*, *44*) (e.g., photoplethysmography), acoustic (*45*, *46*) (e.g., ultrasound echo tracking), and mechanical sensor (*47*–*49*) (e.g., piezoelectric sensor), our approach achieved a fully flexible, stretchable, and self-powered piezoionic sensor that directly transduces arterial pulses at the wrist into electrical signals. The combination of these features enables seamless, skin-conformal, and self-powered sensing for BP monitoring. To verify the capability for physiological signal acquisition, the frequency-dependent piezoionic response of the PP25/IL sensor was characterized within the typical pulse frequency range of 1 to 3 Hz (corresponding to heart rates of 60 to 180 bpm), as shown in fig. S16. The PP25/IL sensor exhibited a stable and continuous output voltage at all test frequencies, demonstrating its reliability in dynamic physiological signal acquisition. As shown in fig. S17, the in vivo histopathological evaluation confirmed that the ionogel patch elicited no accumulation of inflammatory cells (e.g., neutrophils), indicating no noticeable skin irritation or acute toxicity after a 7-day contact period.

High-accuracy continuous BP analysis is ensured by two aspects. On the one hand, PP25/IL sensor is highly accurate to detect small variations in the wrist arterial pulse waveform and exhibits high stability with negligible baseline drift and good stretchability for user comfort. On the other hand, we designed a physics-informed machine learning algorithm to process sensory data. The algorithm exploited the time-frequency power spectrum, whose main advantage lies in the interpretability of features. These features, which capture essential physiological information from the waveform data, were then fed into the machine learning model, significantly enhancing the prediction performance and reliability of our continuous BP-monitoring system.

The algorithm consists of three functional modules, including (i) time-frequency domain multimodal feature extraction, (ii) extreme gradient boosting (XGBoost) model machine learning, and (iii) ensemble classification. The pulse wave signals from the human body are collected at the wrist using the PP25/IL sensor, acquired by the signal conditioning circuit and transmitted to the signal processing unit. The continuous pulse wave signal is fed into two core submodules. The first submodule extracts multimodal features in time-frequency domain. The 1D pulse wave signal is first converted into 3D power density spectrum using time-frequency analysis. This step not only adds the frequency dimension to the 1D data but also combines time and frequency information within the power spectrum. By fusing time and frequency, it substantially improves the interpretability of the features extracted, unlike a conventional Fourier transform, which treats time and frequency independently. Then, the resulting time-frequency power spectrum is analyzed in two ways. First, for frequency power density analysis, the differences in frequency components and power density of the pulse wave signals among volunteers are examined, and their relationships with variations in BP are evaluated. Second, for time-frequency analysis, a time-domain window is applied to track the dynamic changes in the frequency components of the pulse wave over time. These dynamic patterns are then aligned in time with the corresponding BP measurements. Additionally, the second submodule incorporates demographic information (age, sex, height, weight, and body mass index). By combining the extracted signal features with these demographic data, a vascular tension feature is derived to enhance the model’s prediction accuracy.

The sensing data and vascular tension features are then fed into the second module. It enables the prediction model to effectively select important features and adjust their weights, ultimately producing the estimated BP values. Given that the BP sensing system is intended for routine use by the general population and the available dataset remains relatively limited in scale, the use of complex models such as deep neural networks poses a substantial risk of overfitting. To mitigate this risk, ensemble learning techniques are used. Specifically, XGBoost is adopted as the core predictive model. To identify the optimal model, we further evaluated several machine learning models, including adaptive boosting, support vector regression, and the *K*-nearest neighbor algorithm. The evaluation results indicated that the XGBoost model achieved the highest accuracy among all tested models, as shown in fig. S18. XGBoost (*50*), an advanced and optimized implementation of the gradient boosted tree algorithm, enhances predictive accuracy and model robustness by iteratively combining the outputs of multiple simpler models. For model training and validation, the collected data are preprocessed, randomly shuffled, and split into training and test sets with a ratio of 4:1. The reliability of the model is rigorously evaluated using fivefold cross-validation.

Module 3 is responsible for continuous BP monitoring and result visualization. This module receives window-level regression outputs from module 2 and uses the trained model for inference. The final BP estimate, denoted as $\sum f_{n}\left(x_{i}\right)$, is obtained by computing the weighted sum of outputs from all decision trees within the ensemble. The system continuously generates real-time predictions of SBP, DBP, and MAP, which are then presented to the user interface.

As shown in Fig. 5B and movie S2, the sensor output, which carries the arterial pressure waveform, was acquired from one wrist using the commercial Biopac instrument, serving as reference data. This reference instrument used an inflatable cuff for BP measurements and included a fingertip sensor that provided a reference waveform. The PP25/IL sensor was used to continuously collect arterial pulses from the other wrist. The representative continuous pulse waveforms acquired using the PP25/IL sensor is presented in fig. S19. As shown in Fig. 5C, the PP25/IL sensor successfully captures key features with each pulse cycle (namely, the P1, P2, and P3 peaks). This level of high accuracy and reliability is crucial for subsequent computation for BP.

To gather pulse waveforms for the quantitative analysis of BP, we collected data from 30 volunteers aged 20 to 40 years. Participants were of heights from 155 to 180 cm and body weights from 45 to 80 kg. All wrist measurements were performed under resting conditions. The distribution of BP ranges, with SBP ranging from 113 to 141 mmHg and DBP from 68 to 92 mmHg, among the volunteers participating in the study is illustrated in Fig. 5D.

With the machine learning framework, the achieved accuracy of BP estimation is as follows: the mean absolute error for SBP is 5.89 ± 1.35 mmHg, and the DBP is 3.61 ± 1.13 mmHg. The distribution of predicted BP values, shown in Fig. 5E, closely resembles that of the reference BP values (Fig. 5D) and demonstrates the high sensing accuracy of the PP25/IL sensor. Additionally, as shown in Fig. 5F and fig. S20, comparing the model-estimated BP values with the reference values, the Bland-Altman plot shows mean errors of −1.62/−0.14/−0.63 mmHg for SBP/DBP/MAP, with SDs of 5.95/5.16/4.18 mmHg, respectively. Figure 5G further compares the reference and estimated BP values using violin plots for SBP, DBP, and MAP. The distributions are shown together with the mean and ±1 SD. SBP reference and estimated values were 128.76 ± 6.46 mmHg and 129.10 ± 4.32 mmHg. DBP reference and estimated values were 81.28 ± 6.52 mmHg and 81.42 ± 5.92 mmHg. MAP reference and estimated values were 97.10 ± 5.78 mmHg and 97.31 ± 4.58 mmHg. The estimated distributions closely overlap with the reference distributions in both central tendency and spread. The mean values of estimated BP are in close proximity to their corresponding reference BP values and the comparable SDs, supporting the reliability of the proposed cuffless BP estimation system. The estimation errors were compared to the well-established BHS standard (*51*). As stipulated by this standard, grade A is achieved provided that all of the following accuracy thresholds are met: ≥60% within 5 mmHg, ≥85% within 10 mmHg, and ≥95% within 15 mmHg. As shown in Fig. 5H and table S4, our prototype meets all three requirements simultaneously: 72% within 5 mmHg, 90% within 10 mmHg, and 97% within 15 mmHg, satisfying the grade A rating and demonstrating the feasibility of BP estimation. These results are presented to evaluate the system performance, with a detailed discussion available in note S6.

The time-frequency power spectra of three volunteers with different characteristics are selected analysis specific time-frequency power spectrum features and explore their relationship with BP. For example, the stability of the power spectrum frequency distribution over time is a key indicator of BP health. Figure 5I illustrates three typical states from left to right. In a stable power spectrum, frequency information exhibits minimal variation over time and is generally associated with healthy BP levels. In a relatively unstable state, the frequency information shows moderate fluctuations. In an unstable state, the frequency information varies markedly over time, often correlating with hypertension and an increased risk of cardiovascular disease. Notably, the data from volunteer 3 with pronounced temporal fluctuations indicates instability, serving as an early warning and prediction of emergent pathological conditions. Thus, the power spectra provide an intuitive visual representation from which spectral stability can be directly assessed at a glance, thereby evaluating the user’s potential BP status. This interpretability analysis, which directly links a physical characteristic (i.e., the temporal stability of the time-frequency power spectrum) to core physiological indicators (i.e., BP levels and cardiovascular risk), is crucial not only for the algorithm accuracy but also for interpretability of physiological indicators in the model’s predictions.

To evaluate system stability and long-term reliability, two sets of experiments were conducted. For system stability evaluation, continuous monitoring was performed on a volunteer for up to 600 s, as shown in Fig. 5J. The results show stable noise levels and negligible baseline draft in the system outputs for SBP, DBP, and MAP. Specifically, the mean error for SBP is 5.65 mmHg over the first 100 s and 5.93 mmHg over the last 100 s, indicating reliable performance during continuous monitoring. For long-term stability evaluation, the volunteer was tracked over 7 consecutive days for multiday monitoring, as shown in Fig. 5K. BP-monitoring accuracy remains highly stable throughout the week, satisfying the grade A of BHS standard.

## DISCUSSION

This work presents a hydrogen bond optimization strategy for simultaneously enhancing the mechanical and piezoionic performance of copolymer ionogels. PU is copolymerized with PVA to control the crystallinity and liberate H-bond sites for TFSI^−^ counterion immobilization onto the PVA backbone, which significantly increases the difference in cation-anion migration. After optimization, the resultant ionogel produces a high voltage output of 20 mV, which is a 10-fold improvement, along with exceptional stretchability of 1510% and toughness of 23.1 MJ m^−3^. We also proposed a wearable, noninvasive, and continuous BP analyzer that integrated the piezoionic ionogel sensor with flexible electronic circuits and machine learning algorithm. The physics-informed machine learning framework processes the captured wrist arterial pulse waveform into SBP and DBP to a high accuracy that meet the grade A of the BHS standard. This H-bond optimization approach opens promising avenues for designing high-performance mechanoresponsive piezoionic ionogels and sensors for the wearable, personalized, and intelligent healthcare revolution.

## MATERIALS AND METHODS

### Materials

Short-chain PVA (PVA-1799; degree of hydrolysis, 98 to 99 mol %), poly(propylene glycol) bis(2-aminopropyl ether) with a number-average molar mass (*M*_n_) of 400 g mol^−1^, and [BMIM][TFSI] were purchased from Shanghai Macklin Biochemical Technology Co. Ltd. IPDI, dimethyl sulfoxide (DMSO; ≥99.5%), and dibutyltin dilaurate (DBTDL; ≥95%) were purchased from Shanghai Aladdin Biochemical Technology Co. Ltd. All reagents were used as received unless otherwise noted.

### Preparation of PVA solution

PVA was dissolved in DMSO and stirred at 90°C for 4 hours to prepare a PVA solution at 15 wt % in DMSO.

### Preparation of iso-PU solution

IPDI and poly(propylene glycol) bis(2-aminopropyl ether) at a 5:4 molar ratio were sequentially dissolved in DMSO under stirring, after which one drop of DBTDL was introduced as catalyst. The reaction solution was maintained at 45°C for 24 hours to yield an iso-PU prepolymer solution at 15 wt % in DMSO.

### Preparation of PP solutions

The 15 wt % PVA and 15 wt % iso-PU solution were combined, and one drop of DBTDL was added as catalyst. The ratio of PVA-PU is varied in the range of 90/10, 80/20, 75/25, and 70/30 parts. The reaction was conducted at 90°C for 1 hour and then maintained at 45°C for 24 hours, yielding four PP solutions with varying iso-PU contents.

### Preparation of PVA/IL and PP/IL ionogels

The IL, [BMIM][TFSI], was added to the PP solutions to achieve an overall IL content of 25 wt %. The mixture was stirred at 90°C for 20 min until optically clear. The resulting PP/IL solutions were cast into molds and frozen at −20°C for 8 hours to yield PP/IL ionogels.

The ionogels corresponding to PVA-PU mass ratios of 100/0, 90/10, 80/20, 75/25, and 70/30 were designated PVA/IL, PP10/IL, PP20/IL, PP25/IL, and PP30/IL ionogel, respectively. The PVA/IL sample (without PU) served as the baseline control.

### Material characterizations

XRD analysis was performed on a Bruker Discover 8 diffractometer using Cu Kα radiation (λ = 1.54 Å). DSC curves were collected on a Mettler DSC3 under a nitrogen atmosphere at a heating rate of 10°C/min from 30° to 350°C. SAXS measurement was carried out on a Xenocs Xeuss 3.0 system with X-rays of λ = 1.54 Å, over a *q* range of 0.006 to 0.2 Å^−1^. FTIR spectra were recorded on a PerkinElmer instrument over a wave number range of 400 to 4000 cm^−1^. Samples were cryo-fractured in liquid nitrogen, and the fracture surface morphology was observed by scanning electron microscopy (Thermo Fisher Scientific Apreo 2). TGA curves were obtained on a PerkinElmer 6000 simultaneous thermal analyzer.

The mechanical properties of the samples were tested using a universal testing machine (Instron 68SC). Tensile stress-strain curves and cyclic tensile loading-unloading tests were recorded at a crosshead speed of 50 mm min^−1^, using rectangular specimens of 15 mm by 50 mm with thickness of 1.5 mm and gauge length of 1 mm. Young’s modulus was calculated from the tensile stress-strain curve by performing a linear fit in small-strain elastic regime (5% strain). Monotonic compression tests were performed at 0.5 mm min^−1^, and cyclic compression tests were performed at 50 mm min^−1^. All measurements were conducted under ambient laboratory conditions. The samples were cut into rectangular strips with dimensions of 15 mm by 50 mm and a thickness of 2 mm.

The ionic conductivity of the samples was determined by EIS in a Ti||sample||Ti configuration. Impedance was measured using an electrochemical workstation (PARSTAT2273) with a 10-mV ac perturbation over a frequency range of 0.1 to 1 MHz. All measurements were conducted at room temperature. The conductivity (σ) was calculated from the impedance using σ = *L*/(*R A*), where *L* is the sample thickness, *R* is the real part of the impedance, and *A* is the sample area.

### Electromechanical transduction characterization

The Instron 68SC universal testing machine served as the pressure source, with a loading rate of 100 mm min^−1^. The sample (1.5 mm thick) was placed on a glass slide, and two copper electrodes (1 cm in diameter) were placed between the sample and the slide for piezoionic voltage and current measurements. The electrodes were located at the bottom of the sample to avoid signal interference caused by electrode displacement during loading. A cylindrical indenter with a diameter of 1 cm was used to compress the samples, ensuring that the center of the loading area was aligned with the electrode. Electrical signals were recorded using a Keithley DAQ6510 data acquisition/multimeter system.

### Ethics declarations

All animal experiments were approved by the Animal Research Ethics Committee of City University of Hong Kong (approval no. AN-STA-00001083) and followed the National Institutes of Health’s *Guide for the Care and Use of Laboratory Animals*. For experiments involving human subjects, the study was approved by the Clinical Research Ethics Committee of the Hong Kong Science and Technology Parks Corporation (approval no. 2023-005). Informed consent was obtained for studies on humans after the nature and possible consequences of the studies are explained.

## Supplementary Materials

### The PDF file includes:

Supplementary Text Notes S1 to S8 Figs. S1 to S20 Tables S1 to S4 Legends for movies S1 and S2 References

## References

[1] W. Tang, Q. Sun, Z. L. Wang, Self-powered sensing in wearable electronics—A paradigm shift technology. Chem. Rev. 123, 12105–12134 (2023). 37871288 10.1021/acs.chemrev.3c00305 PMC10636741

[2] Q. Wang, H. Guan, C. Wang, P. Lei, H. Sheng, H. Bi, J. Hu, C. Guo, Y. Mao, J. Yuan, M. Shao, Z. Jin, J. Li, W. Lan, A wireless, self-powered smart insole for gait monitoring and recognition via nonlinear synergistic pressure sensing. Sci. Adv. 11, eadu1598 (2025). 40238890 10.1126/sciadv.adu1598 PMC12002114

[3] Z. Lai, J. Xu, C. R. Bowen, S. Zhou, Self-powered and self-sensing devices based on human motion. Joule 6, 1501–1565 (2022).

[4] Y. Zhou, X. Xiao, G. Chen, X. Zhao, J. Chen, Self-powered sensing technologies for human Metaverse interfacing. Joule 6, 1381–1389 (2022).

[5] M. T. Chorsi, E. J. Curry, H. T. Chorsi, R. Das, J. Baroody, P. K. Purohit, H. Ilies, T. D. Nguyen, Piezoelectric biomaterials for sensors and actuators. Adv. Mater. 31, e1802084 (2019). 30294947 10.1002/adma.201802084

[6] S. Pan, Z. Zhang, Fundamental theories and basic principles of triboelectric effect: A review. Friction 7, 2–17 (2019).

[7] W. Tang, Y. Wang, G. Xiang, X. Zhao, Z. Pan, Y. Wang, Y. Yang, Y. Wang, G. Yuan, Enhanced high-power performance of Fe-doped PZMNZT piezoelectric ceramics. J. Am. Ceram. Soc. 106, 6868–6878 (2023).

[8] Y. Yan, L. D. Geng, H. Liu, H. Leng, X. Li, Y. U. Wang, S. Priya, Near-ideal electromechanical coupling in textured piezoelectric ceramics. Nat. Commun. 13, 3565 (2022). 35732653 10.1038/s41467-022-31165-y PMC9217974

[9] X. Zhang, Z. Li, W. Du, Y. Zhao, W. Wang, L. Pang, L. Chen, A. Yu, J. Zhai, Self-powered triboelectric-mechanoluminescent electronic skin for detecting and differentiating multiple mechanical stimuli. Nano Energy 96, 107115 (2022).

[10] H. Xiang, L. Peng, Q. Yang, Z. L. Wang, X. Cao, Triboelectric nanogenerator for high-entropy energy, self-powered sensors, and popular education. Sci. Adv. 10, eads2291 (2024). 39612344 10.1126/sciadv.ads2291 PMC11606449

[11] W.-G. Kim, D.-W. Kim, I.-W. Tcho, J.-K. Kim, M.-S. Kim, Y.-K. Choi, Triboelectric nanogenerator: Structure, mechanism, and applications. ACS Nano 15, 258–287 (2021). 33427457 10.1021/acsnano.0c09803

[12] K. Chen, D. Ho, Piezoionics: Mechanical-to-ionic transduction for sensing, biointerface, and energy harvesting. Aggregate 5, e425 (2024).

[13] D. Ho, The piezoionic effect: Biomimetic transduction mechanism for sensing, actuation, interface, and energy harvesting. ChemElectroChem 11, e202300268 (2024).

[14] E. K. Boahen, B. Pan, H. Kweon, J. S. Kim, H. Choi, Z. Kong, D. J. Kim, J. Zhu, W. B. Ying, K. J. Lee, D. H. Kim, Ultrafast, autonomous self-healable iontronic skin exhibiting piezo-ionic dynamics. Nat. Commun. 13, 7699 (2022). 36509757 10.1038/s41467-022-35434-8 PMC9744819

[15] Z. Fang, L. Wang, C. Liang, J. Qiu, T. Zhang, D. Xu, D. Qi, S. Luan, H. Shi, Bioinspired ionic elastomer with skin-like piezo-ionic dynamics enabled by a self-confined multi-interaction network. Adv. Funct. Mater. 35, 2418583 (2025).

[16] J. I. Lee, H. Choi, S. H. Kong, S. Park, D. Park, J. S. Kim, S. H. Kwon, J. Kim, S. H. Choi, S. G. Lee, D. H. Kim, M. S. Kang, Visco-poroelastic electrochemiluminescence skin with piezo-ionic effect. Adv. Mater. 33, 2100321 (2021). 10.1002/adma.202100321 34060148

[17] Y. Li, H. Yin, C. Jiang, Y. Chen, Q. Li, B. Li, Y. Guo, Rapid fabrication of antifreezing piezoionic hydrogels for fast charging and enhanced energy harvesting frequency via ion-regulating nanoparticles. Adv. Funct. Mater. 10, e15804 (2025).

[18] T. Xu, L. Jin, Y. Ao, J. Zhang, Y. Sun, S. Wang, Y. Qu, L. Huang, T. Yang, W. Deng, W. Yang, All-polymer piezo-ionic-electric electronics. Nat. Commun. 15, 10876 (2024). 39738024 10.1038/s41467-024-55177-y PMC11686271

[19] S. Wang, T. Yang, D. Zhang, Q. Hua, Y. Zhao, Unveiling gating behavior in piezoionic effect: Toward neuromimetic tactile sensing. Adv. Mater. 36, e2405391 (2024). 39056155 10.1002/adma.202405391

[20] Y. Dobashi, D. Yao, Y. Petel, T. N. Nguyen, M. S. Sarwar, Y. Thabet, C. L. W. Ng, E. S. Glitz, G. T. M. Nguyen, C. Plesse, F. Vidal, C. A. Michal, J. D. W. Madden, Piezoionic mechanoreceptors: Force-induced current generation in hydrogels. Science 376, 502–507 (2022). 35482868 10.1126/science.aaw1974

[21] W. Zhu, B. Wu, Z. Lei, P. Wu, Piezoionic elastomers by phase and interface engineering for high-performance energy-harvesting ionotronics. Adv. Mater. 36, e2313127 (2024). 38275214 10.1002/adma.202313127

[22] J. Xu, Q. Li, D. Ho, A universal framework for determining the effect of operating parameters on piezoionic voltage generation. Mater. Horiz. 11, 5709–5721 (2024). 39234925 10.1039/d4mh01067a

[23] N. Tang, Y. Jiang, K. Wei, Z. Zheng, H. Zhang, J. Hu, Evolutionary reinforcement of polymer networks: A stepwise-enhanced strategy for ultrarobust eutectogels. Adv. Mater. 36, e2309576 (2024). 37939373 10.1002/adma.202309576

[24] A. Xu, J. Liu, Y. Wang, Z. Xiao, Y. Xie, Leaf-inspired cellulose ionogels under spatial confinement with synergistically enhanced mechanical and sensing performance for flexible sensors. Adv. Funct. Mater. 36, e19010 (2026).

[25] K. Yang, B. Li, Z. Ma, J. Xu, D. Wang, Z. Zeng, D. Ho, Ion-selective mobility differential amplifier: Enhancing pressure-induced voltage response in hydrogels. Angew. Chem. Int. Ed. 64, e202415000 (2025). 10.1002/anie.202415000 39545315

[26] J. Tie, H. Liu, J. Lv, B. Wang, Z. Mao, L. Zhang, Y. Zhong, X. Feng, X. Sui, H. Xu, Multi-responsive, self-healing and adhesive PVA based hydrogels induced by the ultrafast complexation of Fe3+ ions. Soft Matter 15, 7404–7411 (2019). 31465077 10.1039/c9sm01346f

[27] S. Wu, M. Hua, Y. Alsaid, Y. Du, Y. Ma, Y. Zhao, C.-Y. Lo, C. Wang, D. Wu, B. Yao, J. Strzalka, H. Zhou, X. Zhu, X. He, Poly(vinyl alcohol) hydrogels with broad-range tunable mechanical properties via the hofmeister effect. Adv. Mater. 33, e2007829 (2021). 33554414 10.1002/adma.202007829

[28] M. Hua, S. Wu, Y. Ma, Y. Zhao, Z. Chen, I. Frenkel, J. Strzalka, H. Zhou, X. Zhu, X. He, Strong tough hydrogels via the synergy of freeze-casting and salting out. Nature 590, 594–599 (2021). 33627812 10.1038/s41586-021-03212-z

[29] Y. Jiang, N. Tang, X. Wang, D. Pei, H. Zhang, M.-H. Li, J. Hu, Mechanically robust eutectogels enabled by precisely engineered crystalline domains. Nat. Commun. 16, 7417 (2025). 40790118 10.1038/s41467-025-62742-6 PMC12340020

[30] W. Zhao, Y. Zhang, X. Zhao, B. Yu, L. Zhang, S. Ma, F. Zhou, Superior anti-swelling and durably lubricious bio-hydrogels via robust crystalline domain construction for diverse biodevice coating. Matter 8, 102317 (2025).

[31] Y. Li, Y. Li, R. Cao, J. Xie, W. Chen, Amplifying the mechanical resilience of chemically cross-linked poly(vinyl alcohol) films with the addition of boric acid. Macromolecules 57, 6321–6332 (2024).

[32] S. Yin, Y. Ding, Y. Zhang, C. Zhou, C. Qian, G. Che, C. Zhao, L. Jiang, Superstrong and transparent hydrogels with homogeneous multiple networks. Macromolecules 58, 4357–4364 (2025).

[33] L. Xu, Y. Qiao, D. Qiu, Coordinatively stiffen and toughen hydrogels with adaptable crystal-domain cross-linking. Adv. Mater. 35, e2209913 (2023). 36628947 10.1002/adma.202209913

[34] H. Zhuo, X. Dong, Q. Liu, L. Hong, Z. Zhang, S. Long, W. Zhai, Bamboo-inspired ultra-strong nanofiber-reinforced composite hydrogels. Nat. Commun. 16, 980 (2025). 39856088 10.1038/s41467-025-56340-9 PMC11761455

[35] Z. Kong, E. K. Boahen, D. J. Kim, F. Li, J. S. Kim, H. Kweon, S. Y. Kim, H. Choi, J. Zhu, W. Bin Ying, D. H. Kim, Ultrafast underwater self-healing piezo-ionic elastomer via dynamic hydrophobic-hydrolytic domains. Nat. Commun. 15, 2129 (2024). 38459042 10.1038/s41467-024-46334-4 PMC10923942

[36] X. Guan, S. Zheng, J. Luo, X. Liu, X. Wang, B. Zhang, Y. Zhu, D. Li, Q. Han, M. Ueda, M. An, Spider webs-inspired aluminum coordination hydrogel piezoionic sensors for tactile nerve systems. Adv. Funct. Mater. 35, 2414016 (2025).

[37] Q. Wei, C. Zhang, Y. Quan, H. Shi, Y. Zhang, R. Xu, S. Ma, F. Zhou, Enhancing superlubricity and wear resistance in mechanically robust hydrogel via microliter-scale subsurface-initiated polymer brush grafting. Angew. Chem. Int. Ed. Engl. 65, e24676 (2026). 41830205 10.1002/anie.202524676

[38] J. Dai, Y. Xue, X. Chen, Z. Cao, L. Wang, J. Zhang, Y. Zhou, Y. Hu, W. Zhou, W. Tang, X.-Y. Kong, B. Tu, J. Liu, K. Xiao, Artificial nerve for neuromodulation based on structured piezoionic hydrogel. Device 2, 100436 (2024).

[39] D. Wang, Z. Zeng, Z. Ma, B. Li, J. Shao, K. Yang, D. Ho, Soft self-powered solid-state poly(ionic liquid) piezoionic elastomer sensor for extreme temperature operation. Chem. Eng. J. 530, 173003 (2026).

[40] Y. Sun, G. Tian, T. Yang, S. Wang, B. Lan, X. Li, T. Xu, L. Jin, W. Deng, W. Yang, Crosslinking-modulated hydrogel piezoionic sensor for pattern security authentication in human-machine interfaces. Adv. Funct. Mater. 35, 2420187 (2025).

[41] S. G. Chrysant, Relatability of blood pressure monitoring with wearable cuffless devices. Am. J. Cardiol. 169, 145–147 (2022). 35045932 10.1016/j.amjcard.2021.12.028

[42] S. Min, J. An, J. H. Lee, J. H. Kim, D. J. Joe, S. H. Eom, C. D. Yoo, H.-S. Ahn, J.-Y. Hwang, S. Xu, J. A. Rogers, K. J. Lee, Wearable blood pressure sensors for cardiovascular monitoring and machine learning algorithms for blood pressure estimation. Nat. Rev. Cardiol. 22, 629–648 (2025). 39966649 10.1038/s41569-025-01127-0 PMC13200057

[43] Y. Duan, B. Li, K. Yang, Z. Gong, X. Peng, L. He, D. Ho, Ultrahigh energy and power density in Ni–Zn aqueous battery via superoxide-activated three-electron transfer. Nano Micro Lett. 17, 79 (2024). 10.1007/s40820-024-01586-z PMC11607291 39612109

[44] F. Schrumpf, P. Frenzel, C. Aust, G. Osterhoff, M. Fuchs, Assessment of non-invasive blood pressure prediction from PPG and rPPG signals using deep learning. Sensors 21, 6022 (2021). 34577227 10.3390/s21186022 PMC8472879

[45] T. Liu, Y. Mao, H. Dou, W. Zhang, J. Yang, P. Wu, D. Li, X. Mu, Emerging wearable acoustic sensing technologies. Adv. Sci. 12, e2408653 (2025). 10.1002/advs.202408653 PMC11809411 39749384

[46] S. Zhou, G. Park, K. Longardner, M. Lin, B. Qi, X. Yang, X. Gao, H. Huang, X. Chen, Y. Bian, H. Hu, R. S. Wu, W. Yue, M. Li, C. Lu, R. Wang, S. Qin, E. Tasali, T. Karrison, I. Thomas, B. Smarr, E. B. Kistler, B. A. Khiami, I. Litvan, S. Xu, Clinical validation of a wearable ultrasound sensor of blood pressure. Nat. Biomed. Eng. 9, 865–881 (2025). 39567702 10.1038/s41551-024-01279-3

[47] X. Wang, G. Wu, X. Zhang, F. Lv, Z. Yang, X. Nan, Z. Zhang, C. Xue, H. Cheng, L. Gao, Traditional chinese medicine (TCM)-inspired fully printed soft pressure sensor array with self-adaptive pressurization for highly reliable individualized long-term pulse diagnostics. Adv. Mater. 37, e2410312 (2025). 39344553 10.1002/adma.202410312

[48] S. Min, D. H. Kim, D. J. Joe, B. W. Kim, Y. H. Jung, J. H. Lee, B.-Y. Lee, I. Doh, J. An, Y.-N. Youn, B. Joung, C. D. Yoo, H.-S. Ahn, K. J. Lee, Clinical validation of a wearable piezoelectric blood-pressure sensor for continuous health monitoring. Adv. Mater. 35, e2301627 (2023). 36960816 10.1002/adma.202301627

[49] X. Ran, F. Luo, Z. Lin, Z. Zhu, C. Liu, B. Chen, Blood pressure monitoring via double sandwich-structured triboelectric sensors and deep learning models. Nano Res. 15, 5500–5509 (2022).

[50] M. Amjad, I. Ahmad, M. Ahmad, P. Wróblewski, P. Kamiński, U. Amjad, Prediction of pile bearing capacity using XGBoost algorithm: Modeling and performance evaluation. Appl. Sci. 12, 2126 (2022).

[51] J. Li, H. Jia, J. Zhou, X. Huang, L. Xu, S. Jia, Z. Gao, K. Yao, D. Li, B. Zhang, Y. Liu, Y. Huang, Y. Hu, G. Zhao, Z. Xu, J. Li, C. K. Yiu, Y. Gao, M. Wu, Y. Jiao, Q. Zhang, X. Tai, R. H. Chan, Y. Zhang, X. Ma, X. Yu, Thin, soft, wearable system for continuous wireless monitoring of artery blood pressure. Nat. Commun. 14, 5009 (2023). 37591881 10.1038/s41467-023-40763-3 PMC10435523

[52] M. Frisch, G. W. Trucks, H. B. Schlegel, G. E. Scuseria, M. A. Robb, J. R. Cheeseman, G. Scalmani, V. Barone, B. Mennucci, G. A. Petersson, H. Nakatsuji, M. Caricato, X. Li, H. P. Hratchian, A. F. Izmaylov, J. Bloino, G. Zheng, J. L. Sonnenberg, M. Hada, M. Ehara, K. Toyota, R. Fukuda, J. Hasegawa, M. Ishida, T. Nakajima, Y. Honda, O. Kitao, H. Nakai, T. Vreven, J. A. Montgomery, Jr., J. E. Peralta, F. Ogliaro, M. Bearpark, J. J. Heyd, E. Brothers, K. N. Kudin, V. N. Staroverov, T. Keith, R. Kobayashi, J. Normand, K. Raghavachari, A. Rendell, J. C. Burant, S. S. Iyengar, J. Tomasi, M. Cossi, N. Rega, J. M. Millam, M. Klene, J. E. Knox, J. B. Cross, V. Bakken, C. Adamo, J. Jaramillo, R. Gomperts, R. E. Stratmann, O. Yazyev, A. J. Austin, R. Cammi, C. Pomelli, J. W. Ochterski, R. L. Martin, K. Morokuma, V. G. Zakrzewski, G. A. Voth, P. Salvador, J. J. Dannenberg, S. Dapprich, A. D. Daniels, Ö. Farkas, J. B. Foresman, J. V. Ortiz, J. Cioslowski, and D. J. Fox, Gaussian 09, Revision D.01 (Gaussian Inc., 2009).

[53] S. Grimme, J. Antony, S. Ehrlich, H. Krieg, A consistent and accurate ab initio parametrization of density functional dispersion correction (DFT-D) for the 94 elements H-Pu. J. Chem. Phys. 132, 154104 (2010). 20423165 10.1063/1.3382344

[54] F. Weigend, R. Ahlrichs, Balanced basis sets of split valence, triple zeta valence and quadruple zeta valence quality for H to Rn: Design and assessment of accuracy. Phys. Chem. Chem. Phys. 7, 3297–3305 (2005). 16240044 10.1039/b508541a

[55] T. Lu, F. Chen, Multiwfn: A multifunctional wavefunction analyzer. J. Comput. Chem. 33, 580–592 (2012). 22162017 10.1002/jcc.22885

[56] T. Lu, A comprehensive electron wavefunction analysis toolbox for chemists, Multiwfn. J. Chem. Phys. 161, 082503 (2024). 39189657 10.1063/5.0216272

[57] B. Hess, C. Kutzner, D. van der Spoel, E. Lindahl, GROMACS 4: Algorithms for highly efficient, load-balanced, and scalable molecular simulation. J. Chem. Theory Comput. 4, 435–447 (2008). 26620784 10.1021/ct700301q

[58] U. Essmann, L. Perera, M. L. Berkowitz, T. Darden, H. Lee, L. G. Pedersen, A smooth particle mesh Ewald method. J. Chem. Phys. 103, 8577–8593 (1995).

[59] B. Hess, H. Bekker, H. J. C. Berendsen, J. G. E. M. Fraaije, LINCS: A linear constraint solver for molecular simulations. J. Comput. Chem. 18, 1463–1472 (1997).

[60] G. E. Karniadakis, I. G. Kevrekidis, L. Lu, P. Perdikaris, S. Wang, L. Yang, Physics-informed machine learning. Nat. Rev. Phys. 3, 422–440 (2021).

[61] S. Lee, S. Lee, W. Jang, J. Kim, D. K. Yon, J. Lee, Weighted iterative complex demodulation for high-resolution instantaneous frequencies in low-frequency PPG signals from wearable devices. IEEE J. Biomed. Health Inform. 29, 7149–7160 (2025). 40232908 10.1109/JBHI.2025.3561323

[62] T. Chen, C. Guestrin, “XGBoost: A scalable tree boosting system,” in *Proceedings of the 22nd ACM SIGKDD International Conference on Knowledge Discovery and Data* (ACM, 2016), vol. 16, pp. 785–794.

