# Supplementary material for: Piezoionic copolymer ionogel with tunable hydrogen bond enables epidermal blood pressure analyzer
Source: Sci Adv. 2026 Sep 11;12(37):eaee4501. doi: 10.1126/sciadv.aee4501 (PMC13564951; doi:10.1126/sciadv.aee4501)
Supplement: Supplementary file 1 [file sciadv.aee4501_sm.pdf]

Science Advances
AAAS

Supplementary Materials for

Piezoionic copolymer ionogel with tunable hydrogen bond enables epidermal blood pressure analyzer

Xuejie Liu *et al.*

Corresponding author: Derek Ho, derekho@cityu.edu.hk

Sci. Adv. **12**, eaee4501 (2026)
DOI: 10.1126/sciadv.aee4501

The PDF file includes:

Supplementary Text
Notes S1 to S8
Figs. S1 to S20
Tables S1 to S4
Legends for movies S1 and S2
References

Other Supplementary Material for this manuscript includes the following:

Movies S1 and S2

Note 1. Density functional theory (DFT) calculation.

All calculations were carried out with the Gaussian 09 software (*52*). The PBE1PBE functional was adopted for all calculations in combination with the D3BJ dispersion correction (*53*). For geometry optimization and frequency calculations, the 6-311G(d) basis set was used. The singlet point energy calculations were performed with a larger basis set combination, in which the def2-TZVP basis set (*54*) was used. The electrostatic potential is obtained by Multiwfn software (*55*, *56*). The binding energy (E_{bind}) of molecules (M) and TFSI ion was calculated using the formula:

$$E_{\mathrm{bind}} = E_{\mathrm{total}} - E_{\mathrm{(M)}} - E_{\mathrm{(TFSI)}},$$

where E_{total} denotes the DFT total energy of TFSI ion binding to molecule M, $E_{\mathrm{(M)}}$ is the total energy of molecule M and $E_{\mathrm{(TFSI)}}$ is the total energy of $TFSI^-$ ion.

Note 2. Molecular dynamics (MD) simulation.

Atomistic molecular dynamics simulations have been performed in the GROMACS (*57*) (version 2020.6) simulation package, using the general amber force field (GAFF2). The interaction potentials V between atoms is given by:

$$V = V_{\mathrm{bonded}} + V_{\mathrm{nonbonded}}.$$

The bonded terms include the harmonic oscillation of bonds and angles as well as the torsional rotation of dihedrals. The non-bonded term includes the electrostatic interactions between atoms with partial charges as well as the Van der Waals interactions described in terms of the Lennard-Jones 12-6 potentials, given by:

$$V_{\mathrm{bonded}} = \sum_{bonds} \frac{1}{2} k_b (l - l_0)^2 + \sum_{angles} \frac{1}{2} k_\theta (\theta - \theta_0)^2 + \sum_{torsions} k_\phi [1 + cos(n\phi - \phi_0)],$$

and

$$V_{\mathrm{nonbonded}} = \sum_{i=1}^{N} \sum_{j=i+1}^{N} \left\{ \frac{q_i q_i}{4\pi\epsilon_0\epsilon_r r_{ij}} + 4\varepsilon_{ij} \left[\left(\frac{\sigma_{ij}}{r_{ij}} \right)^{12} - \left(\frac{\sigma_{ij}}{r_{ij}} \right)^{6} \right] \right\}.$$

Two systems were simulated, one containing 18 chains of PVA and the other containing 18 PVA-PU chains. Both systems have 132 ion pairs of $BMIM^+$ and $TFSI^-$ and 5425 DMSO solvent molecules. In both systems, the polymer chains were aligned along the x-axis, while all other molecules were randomly distributed within simulation boxes of approximately $12 \times 12 \times 12$ nm and subsequently solvated. After thousands of steps of energy minimization, the systems were equilibrated under the NPT ensemble for 10 ns and followed the production runs of 40 ns under the canonic ensemble. The temperature was coupled to 298 K using the Nose-Hoover method and the pressure was coupled to 1.5 bar using the Parrinello-Rahman method. The cutoff scheme of 1.2 nm was implemented for the non-bonded interactions, and the particle mesh Ewald method (*58*) with a Fourier spacing of 0.1 nm was applied for the long-range electrostatic interactions. All covalent bonds involving hydrogen atoms were constrained using the LINCS algorithm (*59*).

Note 3. Hydrogen-bond-mediated streaming potential model.

The classical streaming potential model is typically formulated for porous materials featuring fixed charged interfaces. When a pressure-driven fluid flows through a channel or a porous medium, the excess counter-ions in the diffuse layer are convected downstream by hydrodynamic forces, giving rise to a streaming current.

Increasing the output voltage in piezoionic gels relies on enlarging the diffusion difference between cations and anions. In the PP/IL ionogel, $TFSI^-$ anions are immobilized on the polymer chains whereas the cations move freely under an applied pressure. This increases the diffusion difference between cations and anions, thereby generates current/voltage signals. This does not

imply that all $TFSI^-$ are permanently fixed; rather, it indicates that a fraction of anions resides in a lower-mobility state. Although the polymer backbone in the PP/IL ionogel is not permanently charged, hydrogen-bond-mediated immobilization of $TFSI^-$ can induce a similar transport asymmetry, which in turn can lead to the generation of streaming potential.

Note 4. Electrocardiogram (ECG) measurement.

First, a Tegaderm transparent film dressing was applied onto the skin as a barrier layer to prevent direct contact with the ionic liquid. No special treatment is required for the skin. Next, a 1×1 cm ionogel patch was placed on top of the Tegaderm film. Finally, a commercial disposable adhesive ECG electrode pad was applied on the outer side of the ionogel to provide mechanical fixation and electrical connection to the Biopac device's leads. ECG recordings were performed under resting and post-exercise conditions.

Note 5. Piezoionic sensing principle for blood pressure estimation.

We utilize piezoionic ionogels to convert subcutaneous pulse pressure into blood pressure (BP) values through biomechanical sensing and pulse wave analysis. The specific process is as follows: During each cardiac cycle, blood flow causes changes in volume and pressure within the underlying blood vessels. These periodic hemodynamic changes transmit radial mechanical stress through the surrounding tissue to the epidermis, resulting in superficial pulse waves. When the piezoionic sensor is closely attached to the skin above an artery (e.g., the radial artery), it actively responds to these subtle mechanical deformations. Mechanical stress effectively converts physiological mechanical stress into a continuous, high-quality piezoionic voltage signal. This signal can accurately reflect the morphological profile of the arterial pulse wave.

Converting the raw voltage signal into BP values (e.g., SBP and DBP) requires analyzing the complex relationship between pulse wave morphology and cardiovascular hemodynamics. Here we use a machine learning algorithm to estimate the BP. First, the raw signal is preprocessed to remove motion artifacts and baseline drift. Then, the proposed algorithm performs feature extraction on the pulse cycle, incorporating time-frequency domain information. According to the principles of pulse wave analysis, the shape of the pulse wave contains useful cardiovascular information. The proposed model is trained to capture morphological and time-frequency features. Based on sensor waveforms and clinical cuff BP references, the model constructs a robust nonlinear mapping. This allows it to accurately convert the characteristics of the collected pressure signals into the continuous BP values.

Note 6. Machine learning model for blood pressure monitoring.

Regarding the operation mechanism, the radial artery pulsation induces subtle displacement and strain on the skin surface, which is transmitted through subcutaneous tissue to the sensor and transduced into a measurable electrical signal. To achieve high accuracy continuous BP estimation, we propose a novel physics-informed machine learning framework based on time-frequency power spectrum. The main advantages of the proposed framework are its physics-informed signal processing, the interpretability of the features, and broad applicability. (*60*) While time-frequency power spectrum methods have been used for continuous BP analysis with optical sensors (e.g., PPG), there is lack of research for continuous pressure pulse wave sensing.

The proposed algorithm works are as follows. Raw continuous pressure pulse wave data is fed into two core modules. Module 1, time-frequency domain power spectrum analysis, extracts multi-modal features in time-frequency domain. The one-dimensional pulse wave signal is first

converted into three-dimensional power density spectrum using time-frequency analysis. This step not only adds the frequency dimension to the 1D data, but also combines time and frequency information within the power spectrum. This is different from traditional methods like Fourier transform analysis that just separate time and frequency. This fusion significantly improves the interpretability of the features extracted. Then, the resulting time-frequency power spectrum are analyzed in two ways. For frequency power density analysis, examining how the frequency components and the power density in pulse wave signals differ across volunteers and relate to differences with BP. For time-frequency analysis, using time-domain window to track how the frequency components of the pulse wave change dynamically over time. These dynamic patterns are aligned in time with the corresponding BP measurements. Additionally, demographic information (e.g., age, sex, height, weight, BMI) is also incorporated. By combining the extracted signal features with this demographic data, we obtain vascular features to boost the model's prediction accuracy. These features are then input into the next module. The features help the prediction model effectively select important features and adjust the weights, finally outputting the estimated BP values. The framework is designed to be general and adaptable to different machine learning or AI algorithms. For Module 2, eXtreme Gradient Boosting (XGBoost) model machine learning, considering that the BP sensing system is intended for everyday users and the current database size is limited, using complex models like deep neural networks carries a high risk of overfitting. To address this, we chose ensemble learning methods. Specifically, XGBoost is used as the core prediction model. XGBoost, an advanced implementation of the Gradient Boosted Tree (GBT) algorithm, significantly improves overall model performance and robustness by combining predictions from multiple simpler models. For Module 3, continuous BP monitoring performs integrated analysis to enable the accuracy of BP measurement. The dataset is divided into training and test sets at a 4:1 ratio, model stability and generalization are assessed with five-fold cross-validation on the training portion, and final performance is reported on the independent held-out test set.

In Module 1, time-frequency domain power spectrum analysis, we apply a Fourier-based time-frequency transform to the i-th sample's discrete pulse waveform $p_i[n]$, where sampling rate is f_s and length is N_i. Thus, the time-frequency complex spectrum is $S_i(t,f)$ and the corresponding power spectral density is $P_i(t,f) = |S_i(t,f)|^2$ (*61*). By this way, the one-dimensional sequence is converted into the three-dimensional time-frequency-power representation. We employ windowed analysis and perform energy normalization to ensure comparability across segments:

$$S_i(t,f) = \sum_{n=0}^{N_i-1} p_i[n] \cdot w[n-t] \cdot e^{-j2\pi f n/f_s},$$

and

$$\widetilde{P_i}(t,f) = \frac{P_i(t,f)}{\sum_{t,f} P_i(t,f)+\epsilon},$$

where $w[\cdot]$ is the analysis window, and ϵ is a constant introduced to prevent numerical instability. This representation provides unified pre-processing data for the two downstream branches in the figure, including frequency power density analysis and time-frequency correlation analysis. In the time-frequency correlation analysis, we select physiologically relevant frequency bands B_k with time information, obtain the frequency band-related energy time series:

$$E_{i,k}(t) = \int_{f\in B_k} P_i(t,f)\,,df.$$

We then construct an inter-band correlation matrix to characterize the signal's co-variation and stability over time. Demographic information collection gathers population attributes such as age, sex, height, weight, and BMI, which serve as covariates reflecting physical priors and inter-individual variability. Finally, these signals are fused into a vascular tension feature representation, thereby improving the robustness and interpretability of the results.

In Module 2, XGBoost model machine learning, the vascular-tension feature from Module 1 serves as the physics-informed input (*62*). The model was constructing by expressing the fused feature for the i-th sample as $x_i = \phi_{\mathrm{TF}}(P_i), \phi_{\mathrm{F}}(\overline{P}_i), \phi_{\mathrm{Corr}}(C_i), \phi_{\mathrm{Time}}(p_i), d_i]$, where $\phi_{\mathrm{TF}}(P_i)$ is the time-varying energy and stability within physiologically relevant bands on the time-frequency power spectrum, $\phi_{\mathrm{F}}(\overline{P}_i)$ is the intensity and distribution of the average power spectral density, $\phi_{\mathrm{Corr}}(C_i)$ reflects inter-band co-variation and coupling, such as the inter-band correlation coefficients and matrix norms, $\phi_{\mathrm{Time}}(p_i)$ is the time-domain morphology and robust statistics, such as the extrema, peak-to-peak amplitude, mean inter-peak interval, and $d_i = [\mathrm{age}_i, \mathrm{sex}_i, \mathrm{height}_i, \mathrm{weight}_i, \mathrm{BMI}_i]$ denotes the demographic covariates. These parameters describe pulse-wave characteristics from complementary physical dimensions. After standardization, they are used as inputs to the SBP and DBP estimation models. XGBoost adopts a stage-wise additive model within the gradient boosting framework, adding regression trees round by round and using first/second-order gradient approximations to minimize a regularized objective. For the output channel $c \in \{\mathrm{SBP}, \mathrm{DBP}\}$, the prediction function is $F_c(x) = \sum_{t=1}^{T} \eta \, , h_{c,t}(x)$, where $h_{c,t}$ is the regression tree added at boosting round t, T is the number of base learners, and η is the learning rate. Training minimizes the regularized empirical risk:

$$\min_{\mathrm{F_c}} \mathcal{L}_c = \sum_{\mathrm{i=1}}^{\mathrm{N}} \ell\left(\mathrm{y_{i,c}}, \mathrm{F_c}(\mathrm{x_i})\right) + \Omega(\mathrm{F_c})\, , d$$

and

$$\Omega(F_c) = \sum_{t=1}^{T} \left(\gamma, J_{c,t} + \frac{\lambda}{2} \sum_{j=1}^{J_{c,t}} w_{c,t,j}^2\right),$$

where $\ell(\cdot)$ is the pointwise loss, $\Omega(\cdot)$ is the structural regularizer, $J_{c,t}$ is the number of leaves in the t-th tree, $w_{c,t,j}$ is the output weight of the j-th leaf, and γ and λ control model complexity, helping to prevent overfitting and promote compact tree structures.

Module 3, continuous BP monitoring, obtains the final results and outputs the continuous BP predictions to the user interface for real-time monitoring. Training and evaluation follow a hold-out plus cross-validation protocol. After unified preprocessing, samples are randomly shuffled and split into training and test sets at a 4:1 ratio. Five-fold cross-validation is conducted on the training set, and final performance is reported on the independent held-out test set, ensuring accuracy, robustness, and interpretability under small-sample conditions.

In wearable BP monitoring, measurement accuracy is still limited by several key factors. Regular calibration and the reliance on relatively stable measurement conditions (e.g., a resting state, a relatively fixed posture, and consistent wearing position and contact) are common limitations of current non-invasive wearable systems. These factors can introduce additional uncertainty across individuals and during daily, dynamic activities. Therefore, the BP monitoring results presented in this work are intended as material- and device-based proof of concept, demonstrating feasibility and potential for future applications.

The sensitivity to sensor positioning, wrist bending, and other contact effects is a critical challenge in the field of wearable continuous BP monitoring, which affects the accuracy of sensing. To mitigate these physical and motion-related influences, we have implemented some approaches

in this work, combining hardware and software processing. For physical securement, we ensure that the sensor is attached to the wrist as securely and consistently as possible. We utilized high-quality medical-grade adhesive tapes. This minimizes relative displacement between the sensor and the skin during minor wrist movements and maintains stable contact pressure. At the software level, the proposed algorithm has preprocessed the raw data. When artifacts are detected, the algorithm can remove the corresponding highly corrupted data segments. Although it may take slightly longer to output an updated BP result during periods of high motion, the adverse effects of noise and artifacts on the final estimation accuracy are significantly mitigated. In addition, we need to acknowledge the objective physiological challenges associated with wrist-based BP measurement. Wrist-based BP sensing might be sensitive to several factors, including the position of the arm relative to heart level. When a subject raises or lowers the forearm, the pressure gradient and local blood-flow conditions change, which may cause appreciable differences in the measured BP. Therefore, measurement errors caused by postural variation and inter-individual physiological differences are important considerations for wrist-wearable BP systems.

Vascular compliance varies over short periods (e.g., vasodilation, vasoconstriction) and long periods (e.g., aging, diabetes), which alters the pressure-signal mapping. While these physiological parameters may influence the signal and cause baseline drift over time, this drift is relatively slow. Such deviations can be compensated for through periodic calibration algorithms, which are standard practice in continuous BP monitoring. Furthermore, long-term factors like aging or diabetes are effectively constant during a single monitoring session and can be accounted for during the initial subject-specific calibration.

Note 7. *In vivo* skin irritation evaluation.

To assess the skin biocompatibility of the ionogel, the dorsal hair of Adult male Sprague-Dawley (SD) rats（n=3） was shaved, and the ionogel samples were applied to the bare skin and secured with 3M adhesive tape for 7 consecutive days. Following the treatment period, the rats were euthanized, and the skin tissues from both the control and ionogel-treated groups were harvested for paraffin embedding and sectioning. The resulting sections were subjected to Hematoxylin and Eosin (H&E) staining to evaluate histopathological changes, such as inflammatory cell infiltration and tissue morphology, under an optical microscope.

Note 8. Safety note for ionogel use.

Although the ionic liquid is physically confined within the polymer network of the ionogel, the on-skin experiments in this study additionally used a Tegaderm transparent dressing as an encapsulation layer to prevent leakage and direct exposure. Moreover, histological examination after a 7-day rat skin-contact study showed no observable skin inflammation compared with the blank control. However, skin irritation may occur if ionic liquids are used without an adequate barrier. Therefore, more comprehensive standardized skin irritation assessments are required for long-term human use and clinical translation. Readers should ensure sufficient encapsulation and follow institutional safety and ethical protocols for any human-subject experiments.

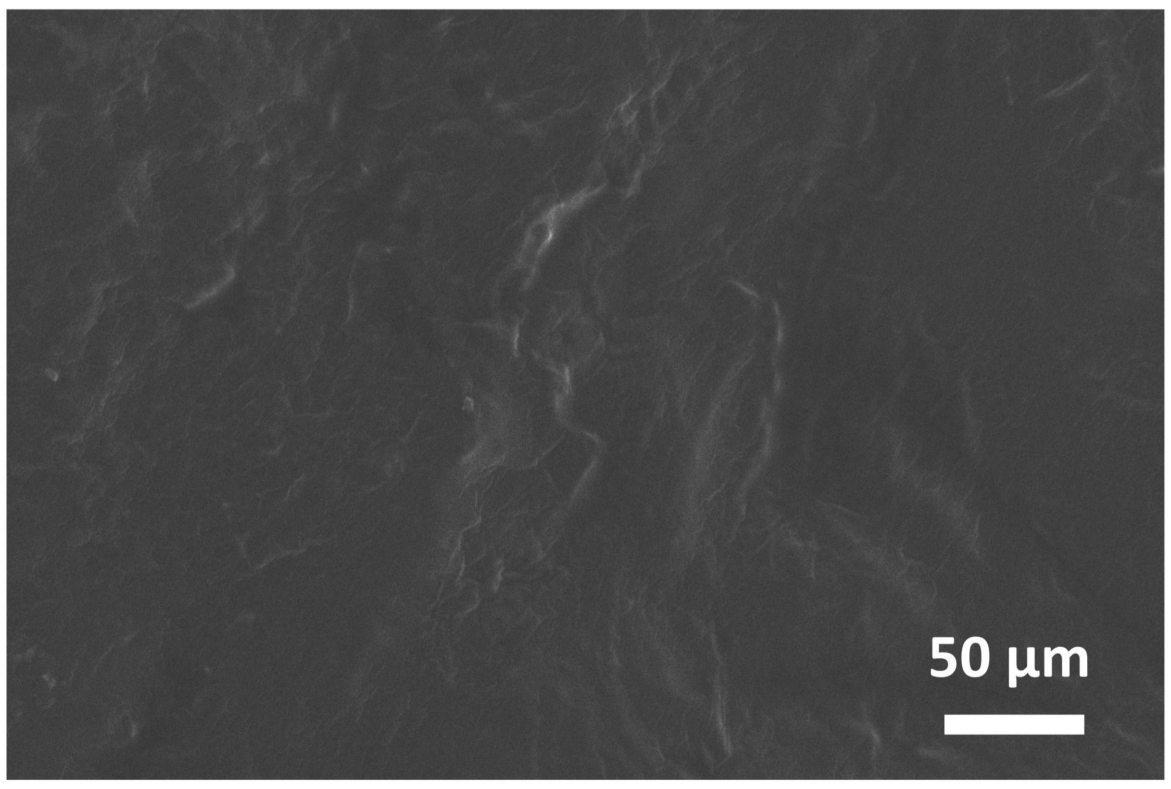

Fig. S1. Cross-sectional SEM image of PP25/IL ionogel.

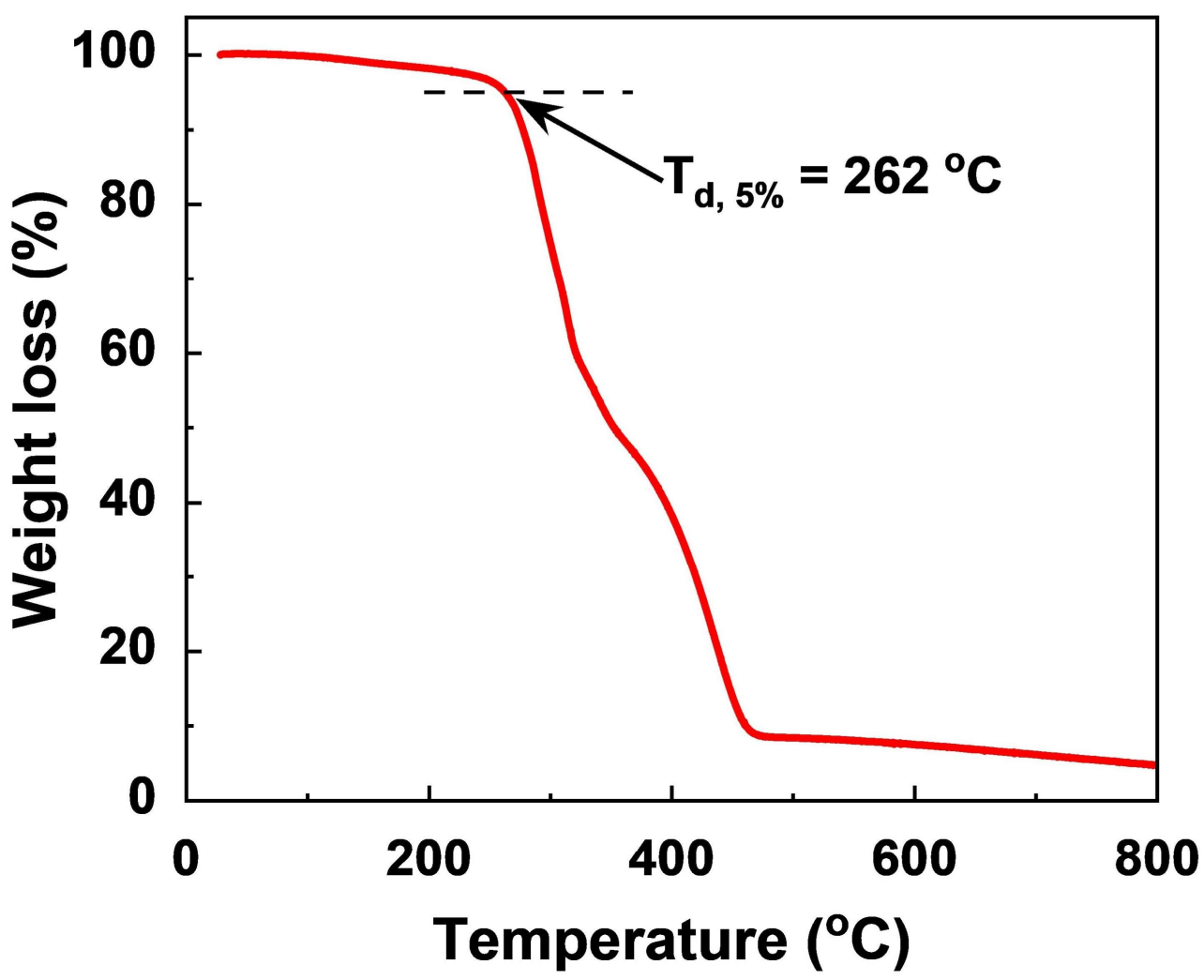

Fig. S2. TGA curve of dry PP25/IL ionogel. The PP25/IL dry ionogel has a $T_{d,\,5\%}$ of 262 °C, with the main decomposition occurring at 250–470 °C, indicating good thermal stability.

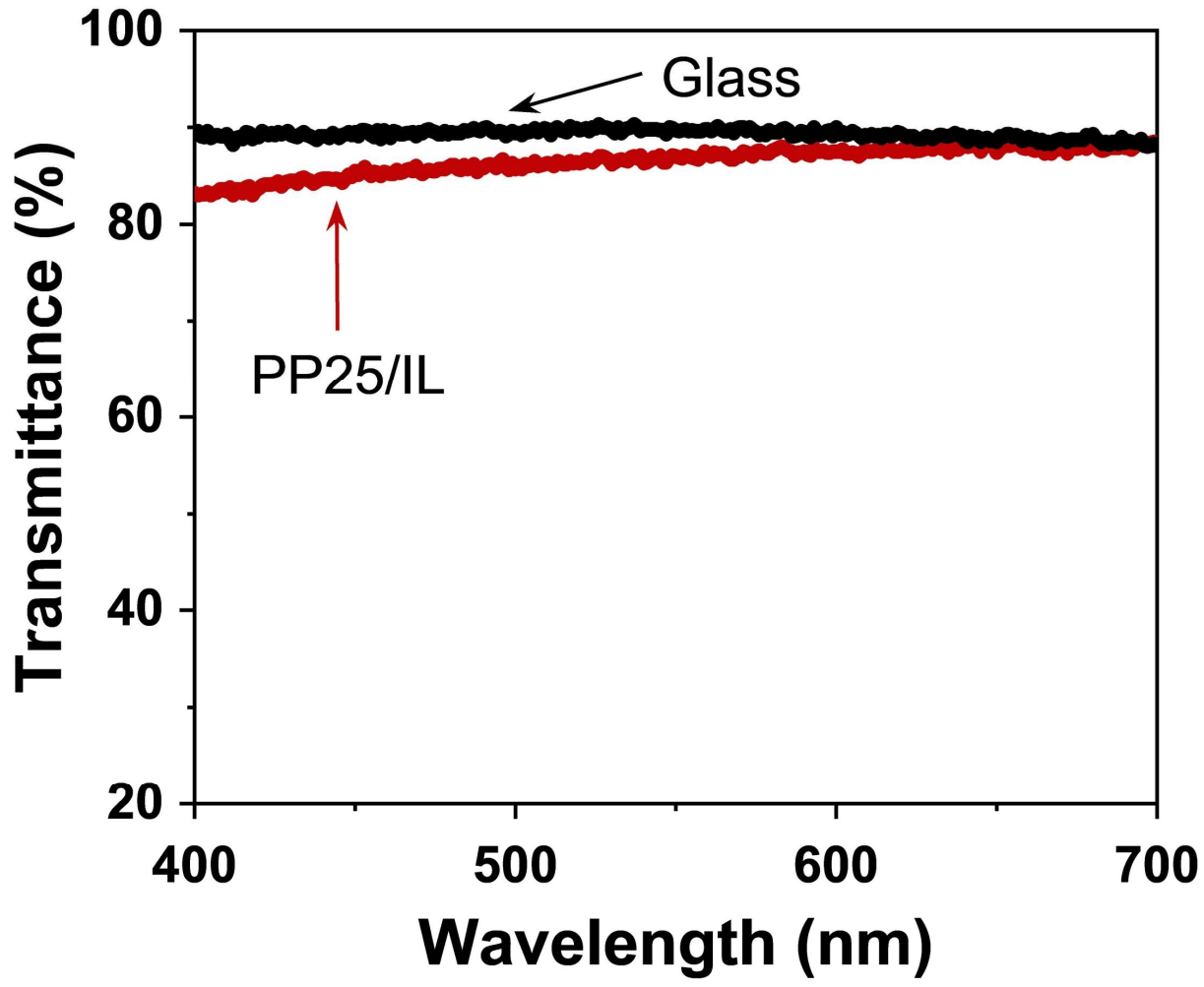

Fig. S3. Transmittance of PP25/IL ionogel and glass. PP25/IL ionogel has a transparency similar to that of glass.

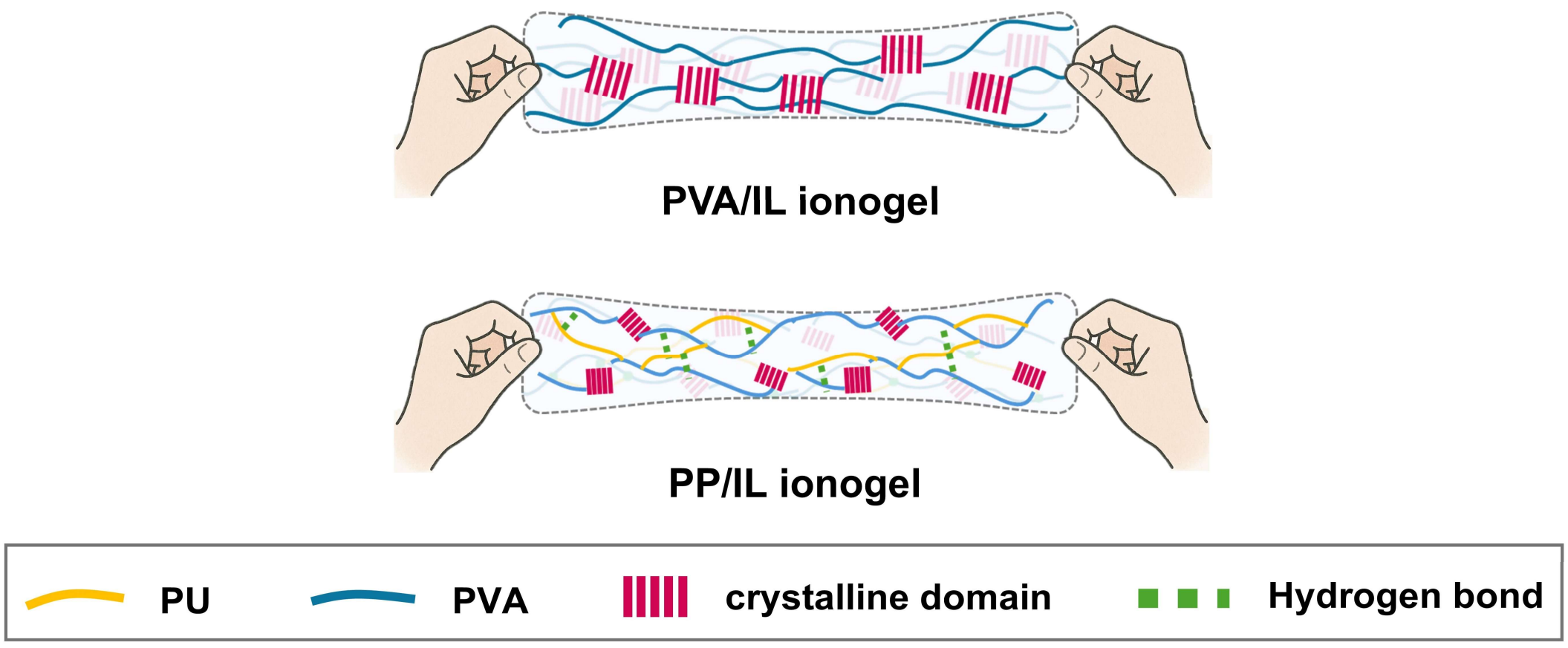

Fig. S4. Schematic diagram of the molecular structures of PVA/IL and PP/IL ionogels under stretching.

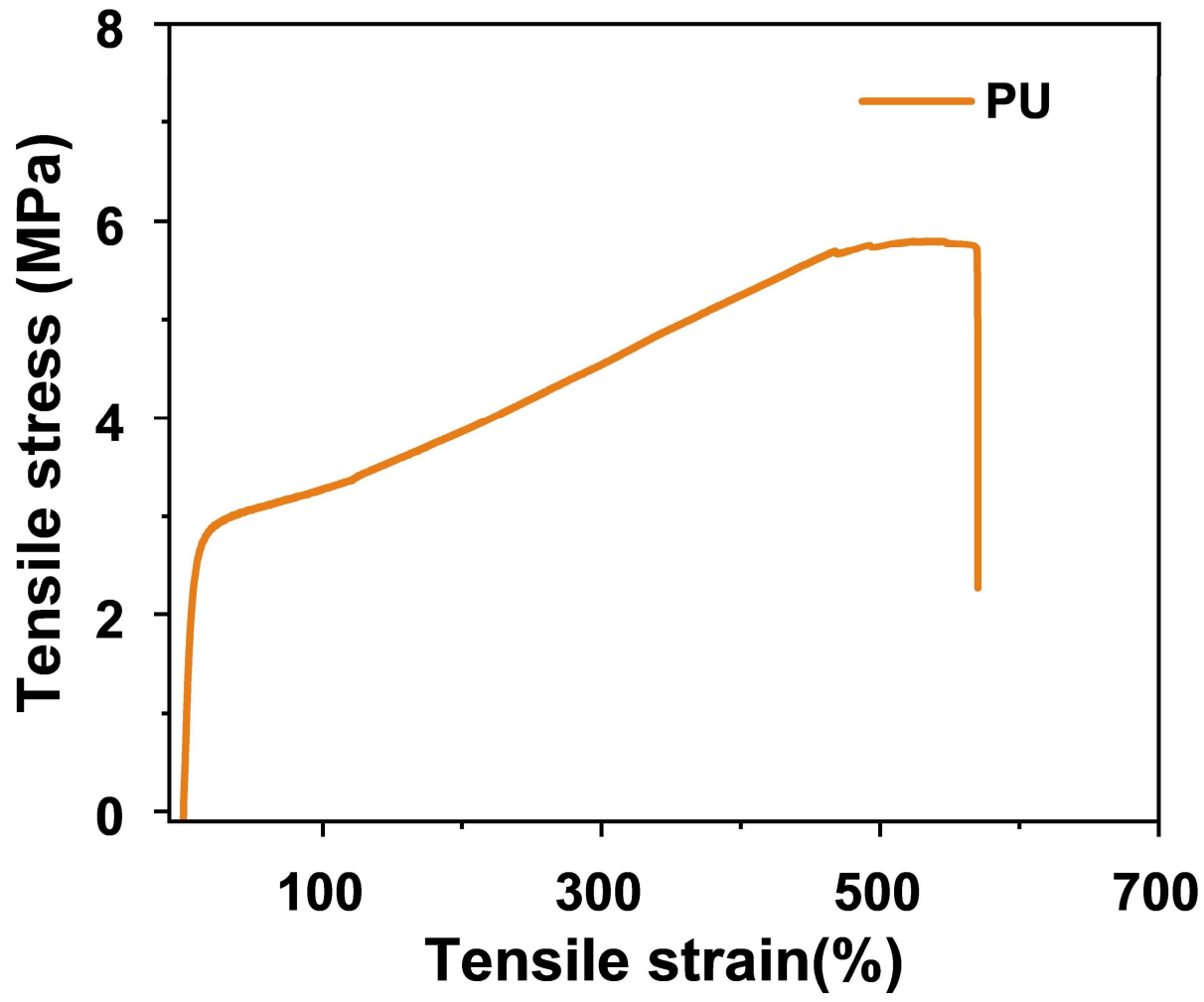

Fig. S5. Stress-strain curve of PU with a Young's modulus of 43.5 MPa.

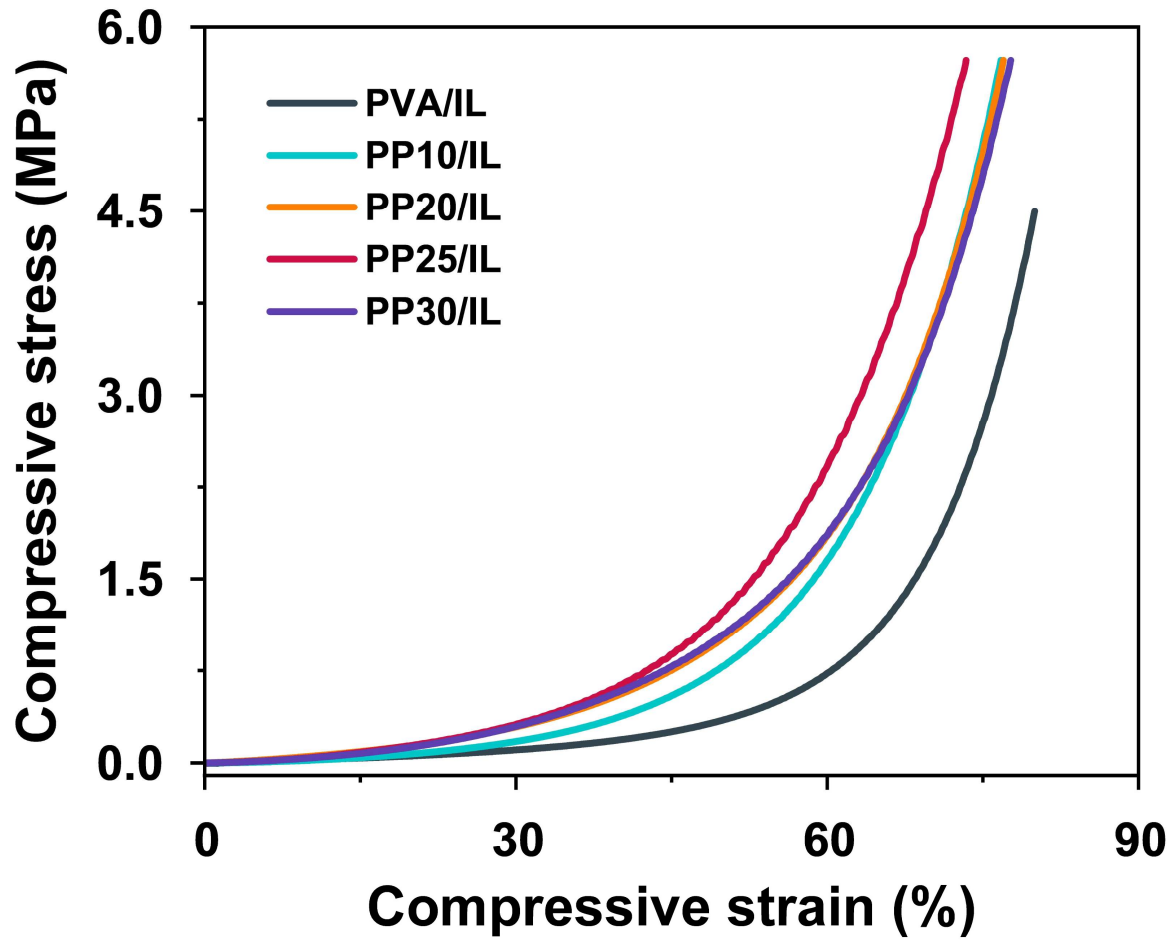

Fig. S6. Compressive stress–strain curves of PP/IL ionogels with different PU contents.

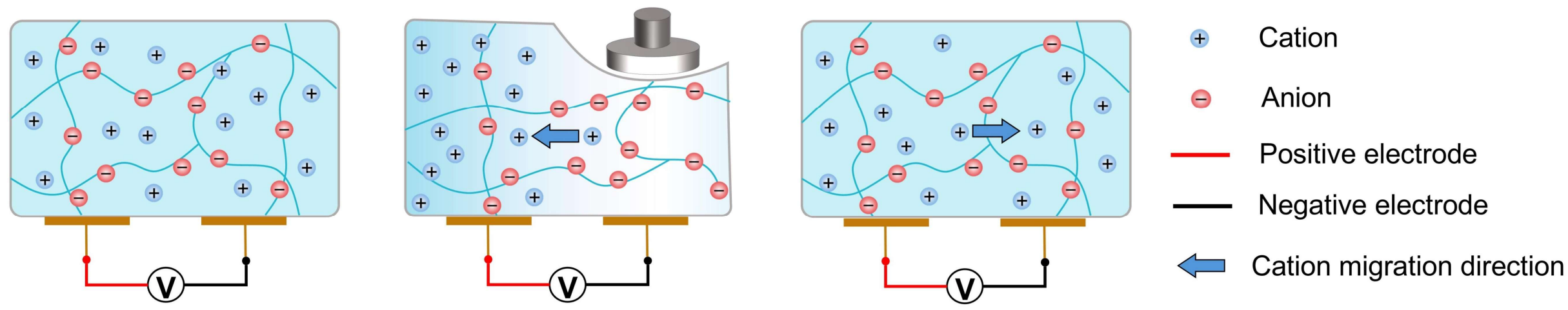

Fig. S7. Schematic diagram of the piezoionic mechanism of ionogels: (A) before, (B) during, (C) after the application of pressure. Before pressure loading, the ions are uniformly distributed throughout the ionogel, resulting in an approximately zero net potential difference. Upon applying pressure, $TFSI^-$ anions are immobilized via hydrogen bonding onto the polymer backbones, whereas the more mobile $BMIM^+$ cations are free to move, therefore redistribute. This asymmetric ionic migration induces a local charge imbalance at the electrode interfaces, thereby generating an open-circuit voltage. After pressure release, elastic recovery and ion diffusion driven by the concentration gradient together cause the voltage signal to decay until the potential difference returns to approximately zero.

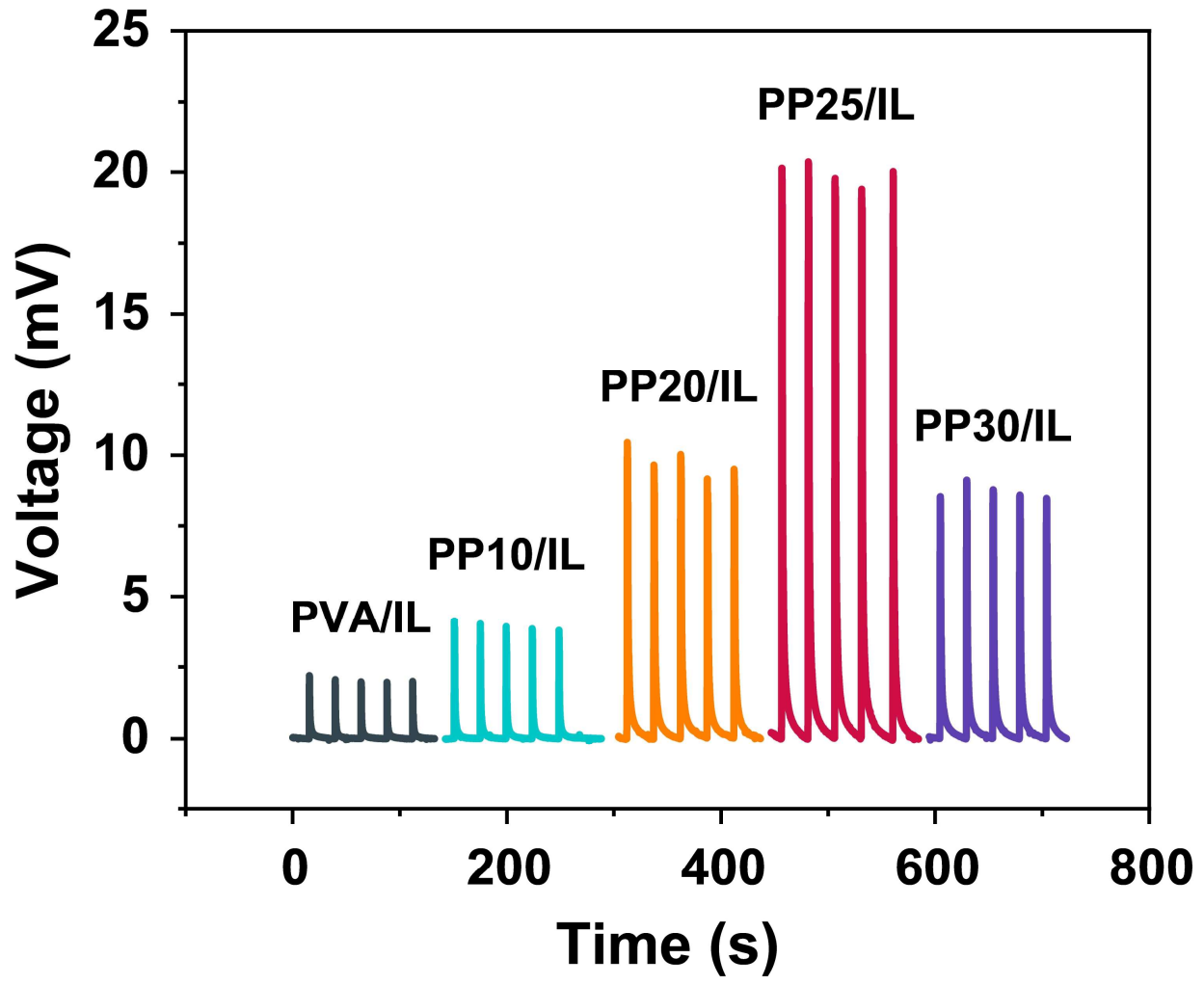

Fig. S8. Voltage responses of PVA/IL and PP/IL ionogels under repeated step compressions at 50 kPa.

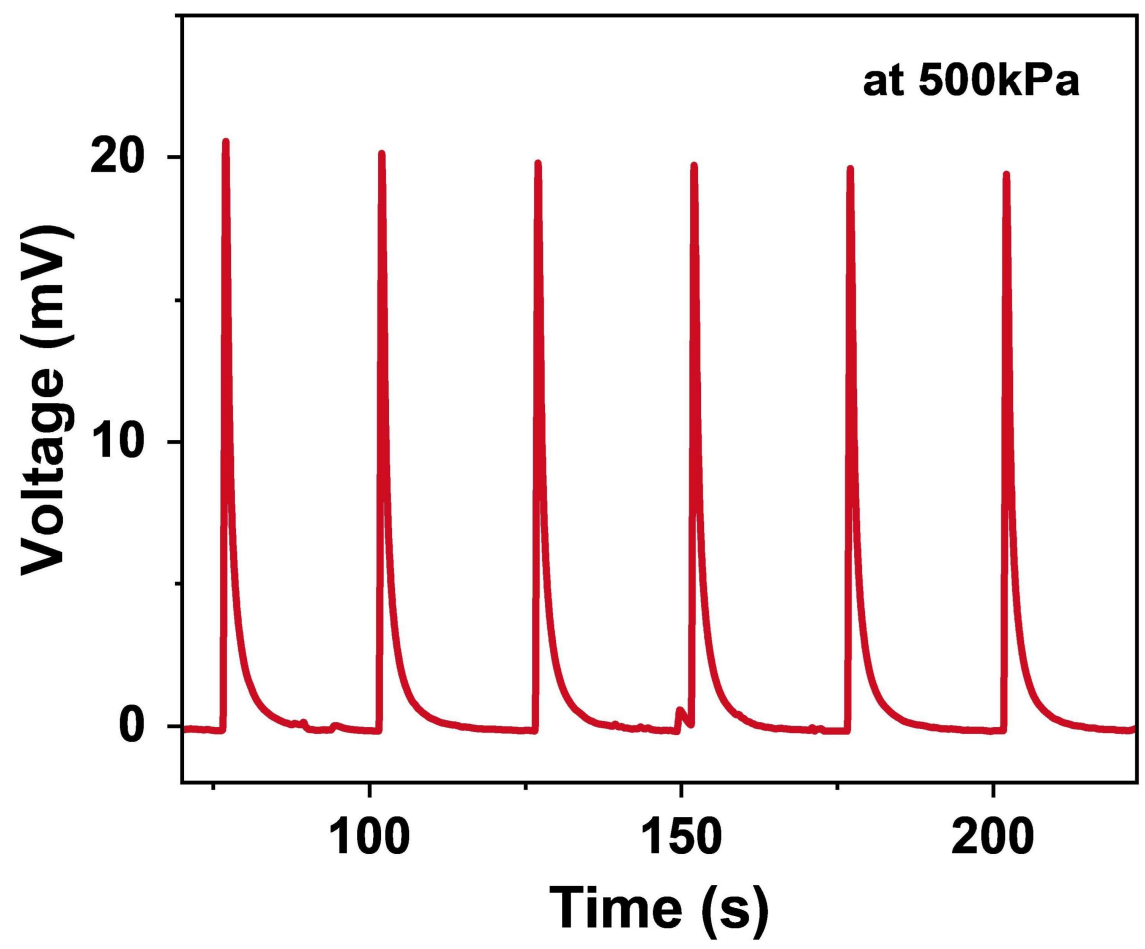

Fig. S9. Voltage response of PP25/IL ionogel at 500 kPa.

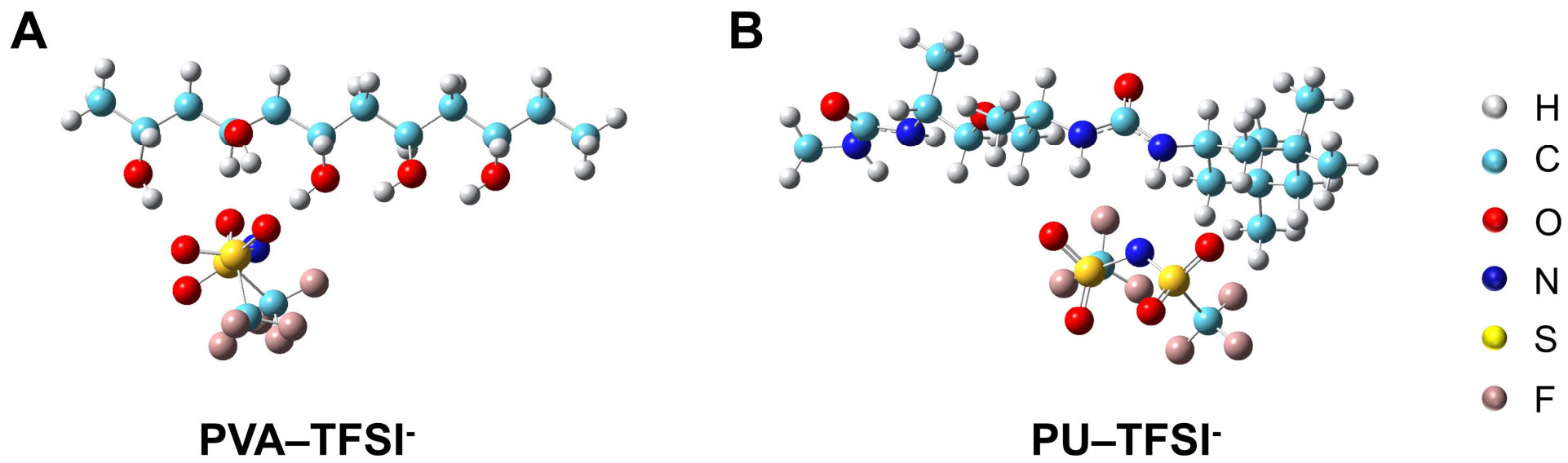

Fig. S10. Molecular models of the (A) PVA–TFSI⁻ and (B) PU–TFSI⁻ used for binding energy calculations.

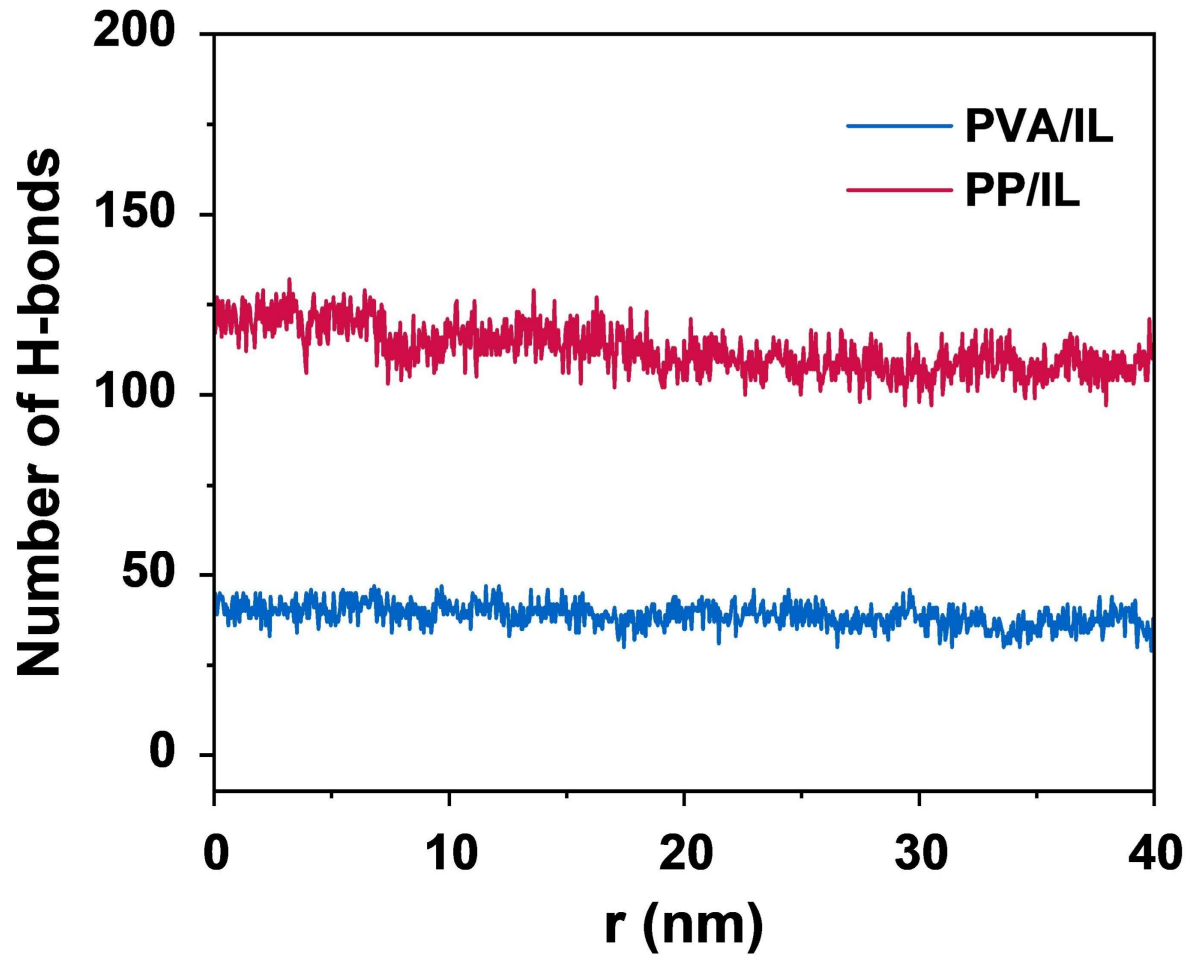

Fig. S11. Number of hydrogen bonds of PVA/IL and PP/IL ionogels.

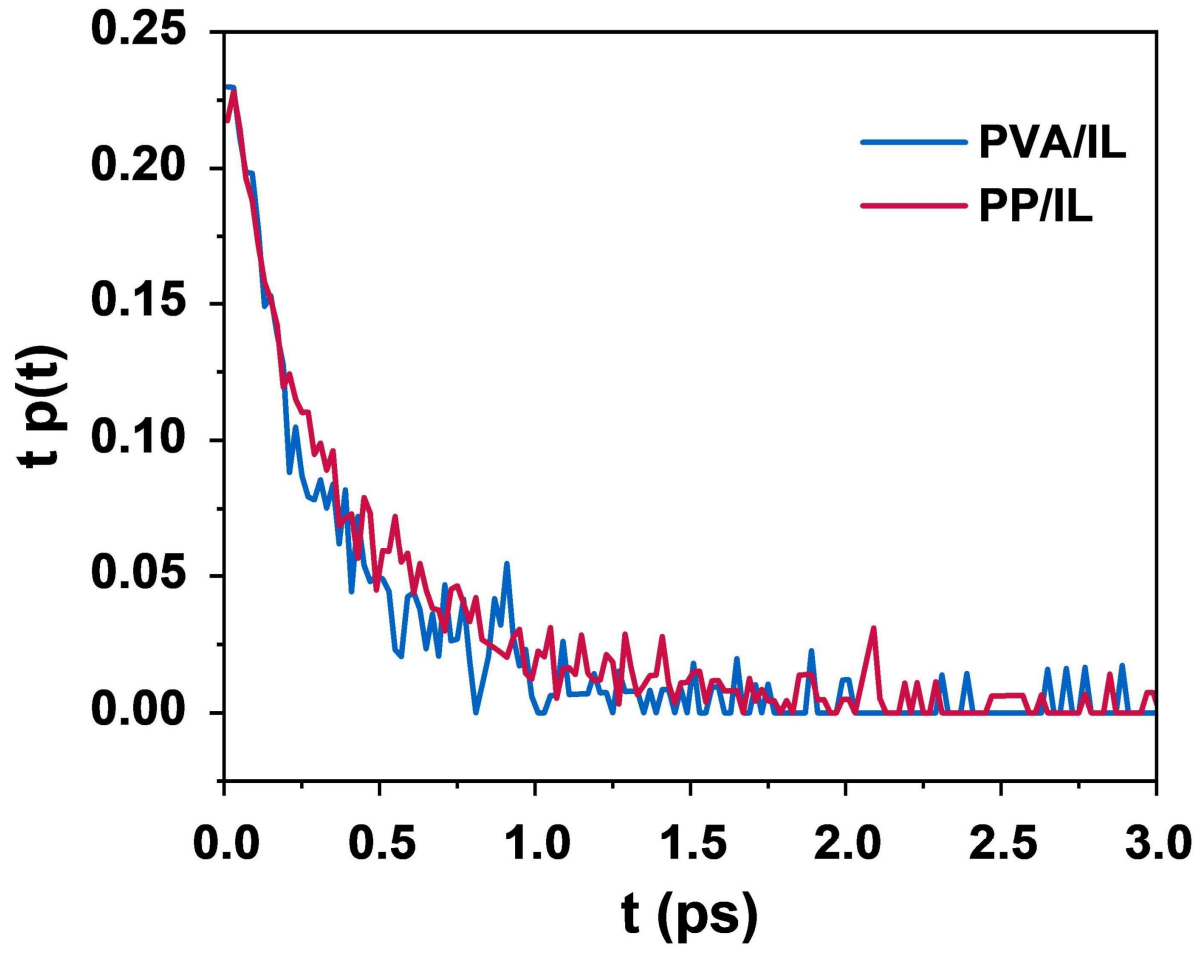

Fig. S12. Hydrogen bond lifetime of PVA/IL and PP/IL ionogels.

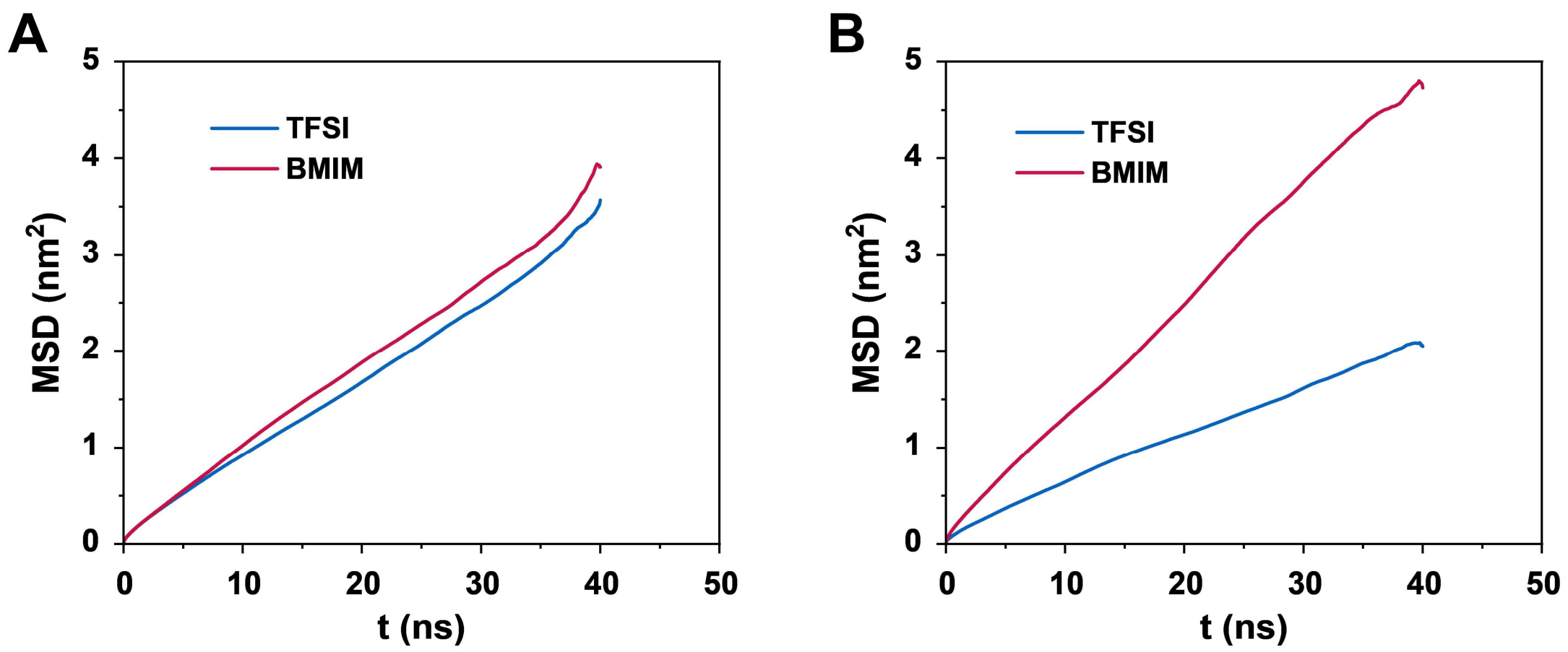

Fig. S13. Mean Squared Displacement of $BMIM^{+}$ and $TFSI^{-}$ ions in (A) PVA/IL ionogel and (B) PP/IL ionogel.

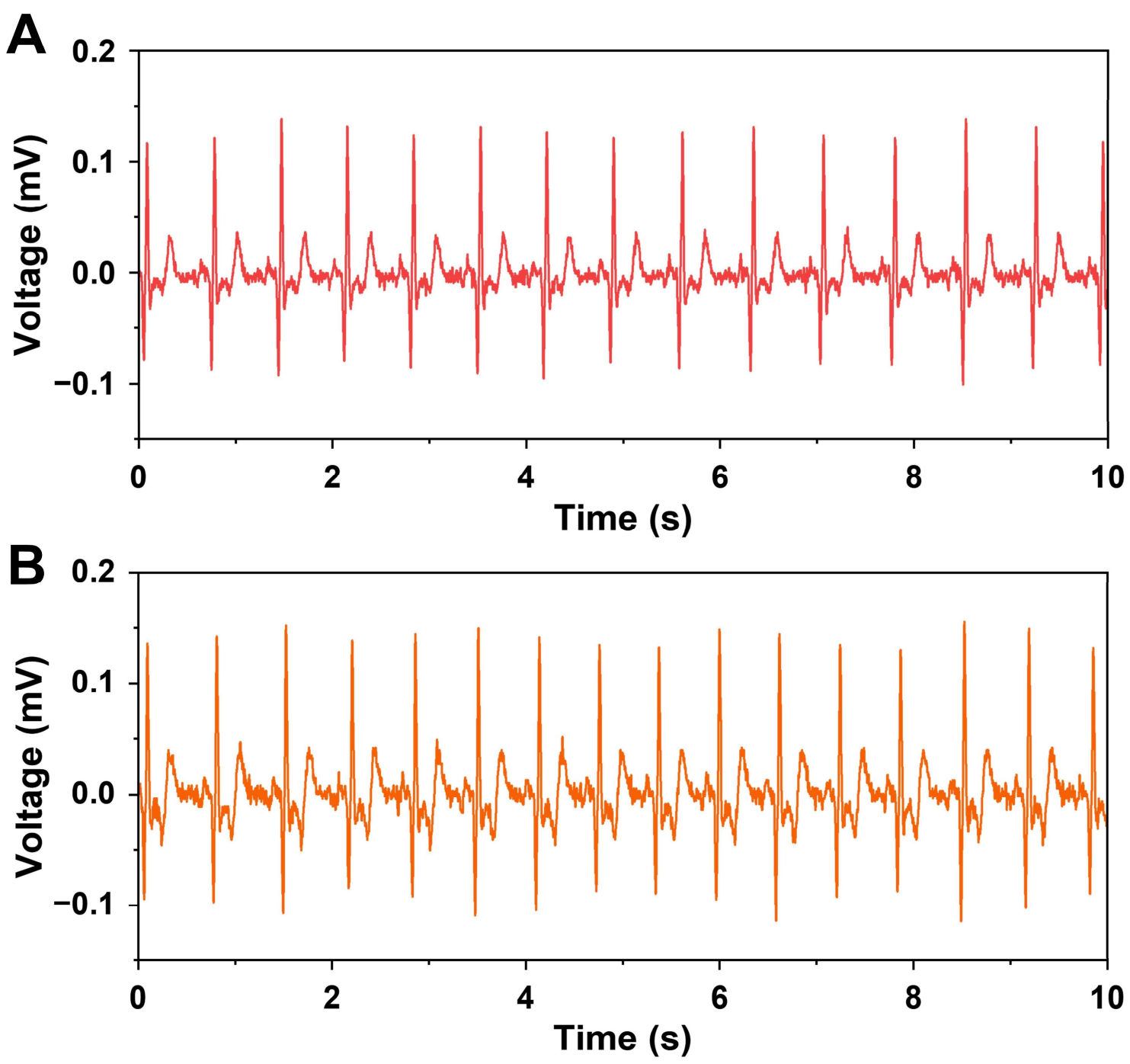

Fig. S14. Electrocardiograms (ECGs) from the same participant: (A) at rest; (B) immediately after a 5-minute jogging. The PP25/IL ionogel electrode provides stable and high-fidelity ECG waveforms. The signal baseline remains stable before and after exercise, with no obvious drift. The ECG recordings show that, compared with the resting heart rate of approximately 85 bpm, the heart rate after exercise increased slightly to about 92 bpm, which is consistent with physiological cardiovascular responses to exercise. The results demonstrate that the PP25/IL ionogel can reliably function as a skin-contact electrode for dynamic ECG monitoring, further highlighting its potential for multimodal, wearable cardiovascular health monitoring beyond pulse and BP sensing.

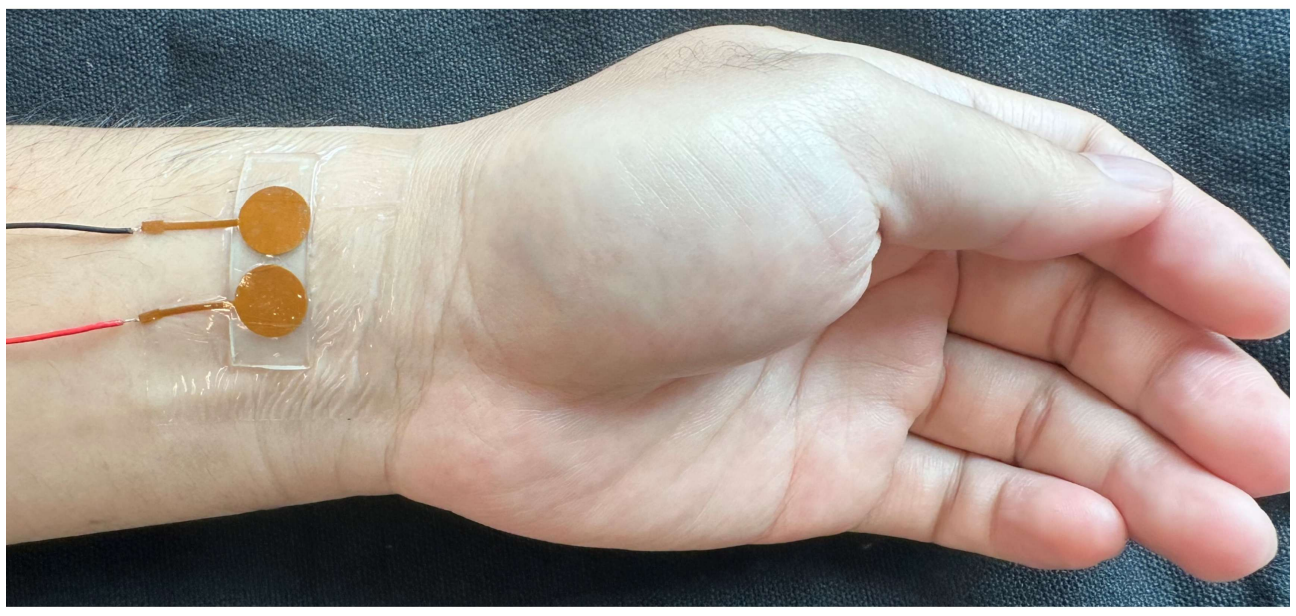

Fig. S15. Optical image of the PP25/IL sensor. Two electrodes were integrated onto the ionogel to form PP25/IL sensor, and the sensor was then placed on the skin over the artery. Medical-grade dressing was used to minimize motion artifacts associated with sensor movement.

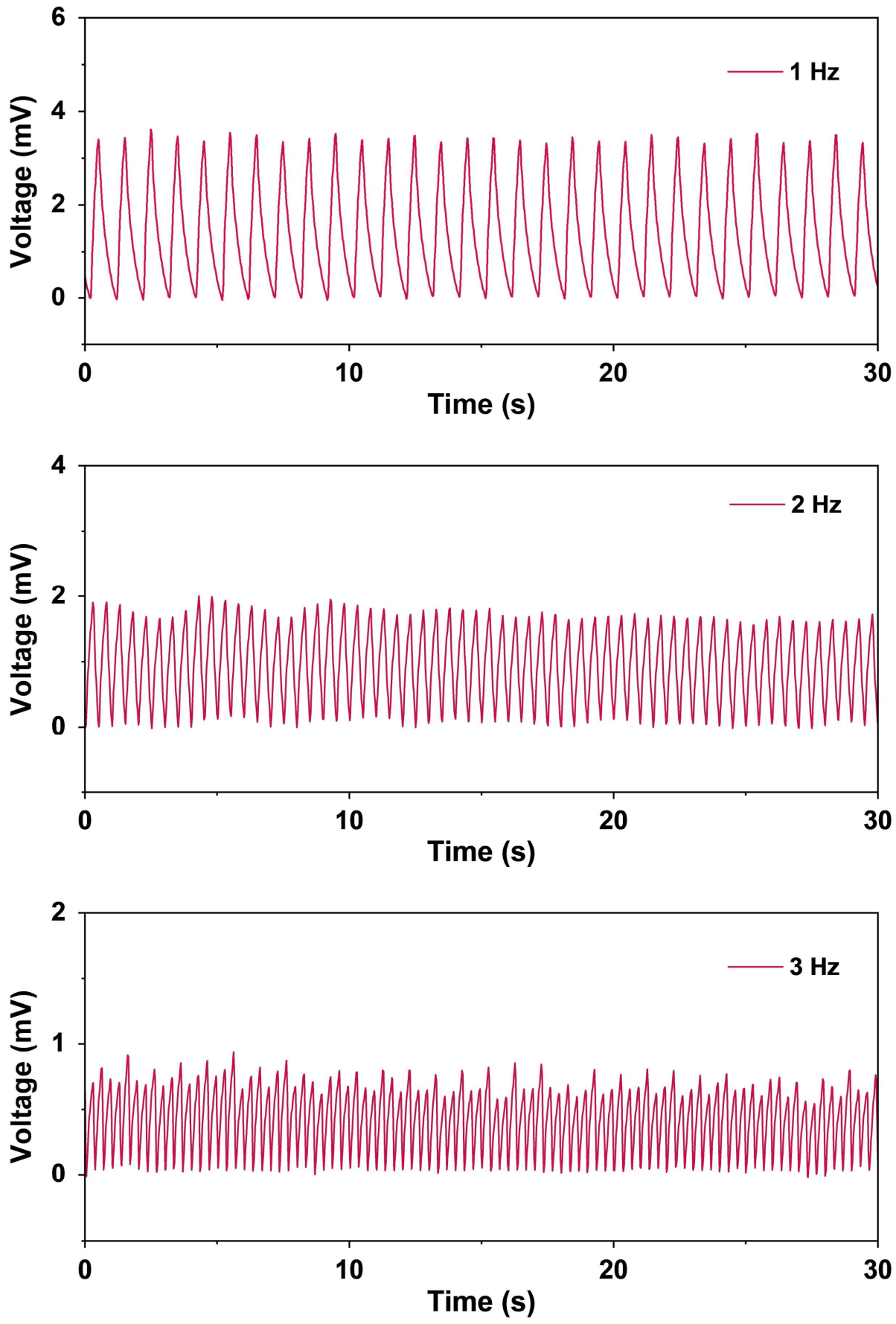

Fig. S16. Piezoionic response at different frequencies. A frequency-dependent behavior in the output voltage magnitude was observed: the peak voltage decreased from ~3.5 mV at 1 Hz to ~1.8 mV at 2 Hz and ~0.6 mV at 3 Hz. This peak decay phenomenon is intrinsically characteristic of piezoionic mechanisms, in which higher loading frequencies prevent sufficient cation migration within each cycle. Despite these frequency-dependent variations, the distinct voltage profiles recorded across the entire 1–3 Hz band demonstrate the capability to operate at various frequencies, thereby validating the sensor's responsiveness and practicality for real-time BP monitoring.

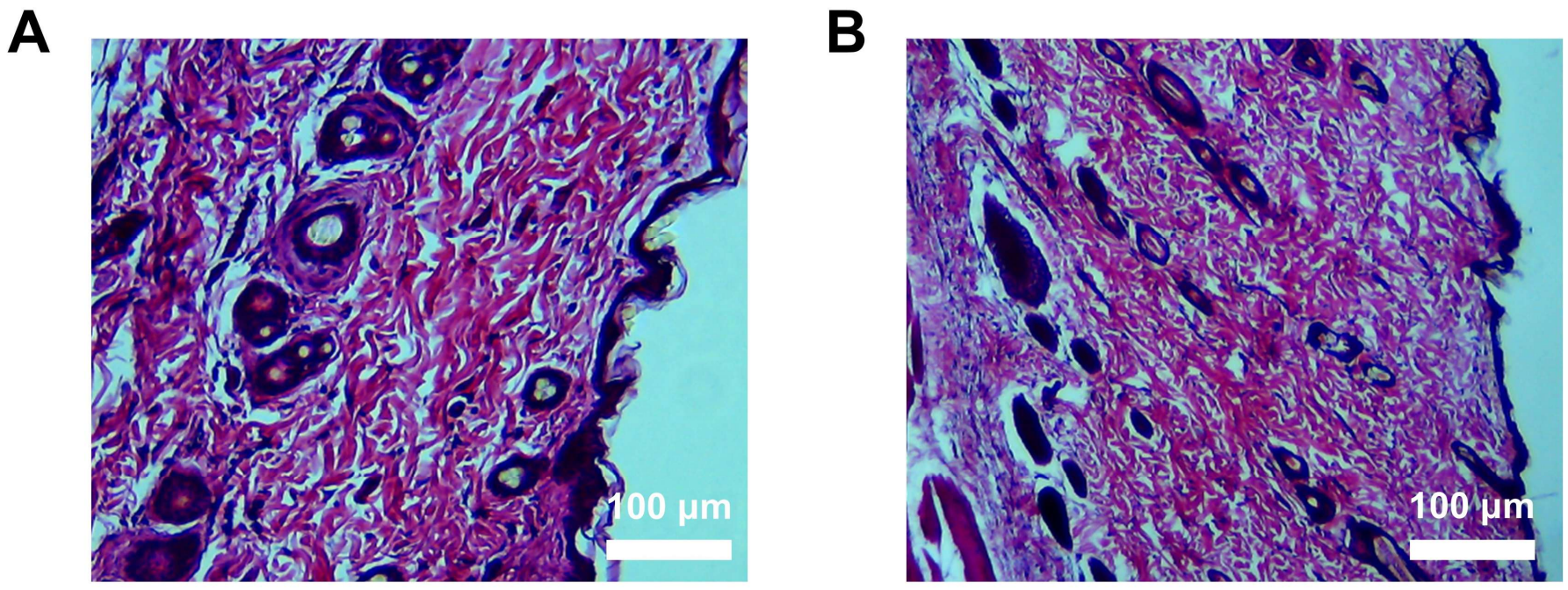

Fig. S17. Hematoxylin-Eosin (H&E) staining skin after 7-day contact with PP25/IL ionogel. (A) control skin and (B) skin exposed to the ionogel patch. Scale bar, 100 μm. The ionogel patchs were applied to the dorsal skin of rats for 7 days, after which the skin tissues were harvested and subjected to H&E staining. No obvious accumulation of inflammatory cells (e.g., neutrophils) was observed compared with the control group. These results indicate that the ionogel does not cause noticeable skin irritation or acute histopathological toxicity.

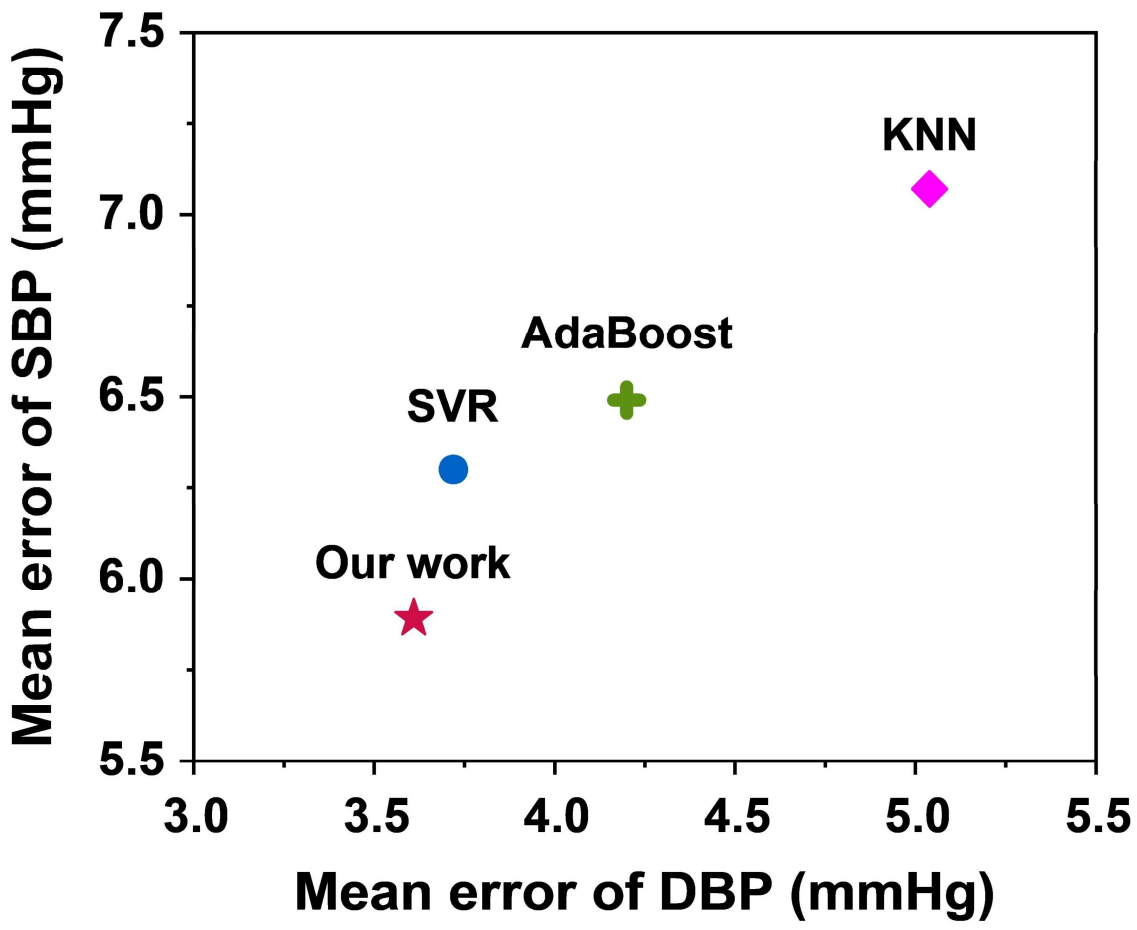

Fig. S18. Comparison of mean errors of SBP and DBP estimation for different algorithms. The algorithms include the method used in this work, support vector regression (SVR), adaptive boosting (Adaboost), and K-nearest neighbor (KNN) algorithm, with mean SBP errors of 5.89, 6.30, 6.49, and 7.07 mmHg and mean DBP errors of 3.61, 3.72, 4.20, and 5.04 mmHg, respectively.

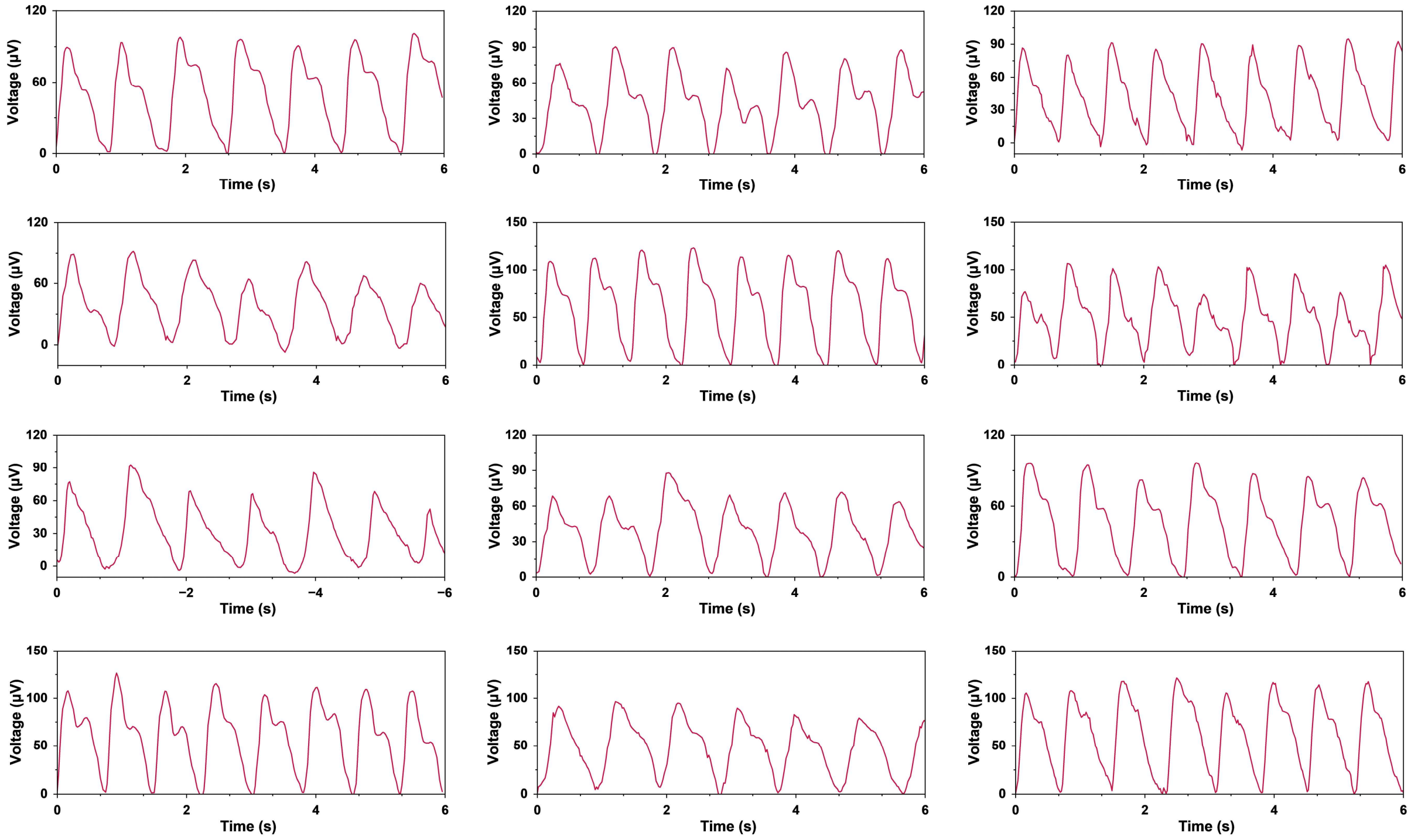

Fig. S19. Pulse waveforms obtained using the PP25/IL sensor. We present the pulse waveforms of 12 participants. The sensor exhibits good reproducibility and stable signal acquisition capabilities across different users. The sensor possesses sufficient sensitivity to accurately capture the physiological features. The ability to distinguish these features is the fundamental prerequisite for reliable BP monitoring.

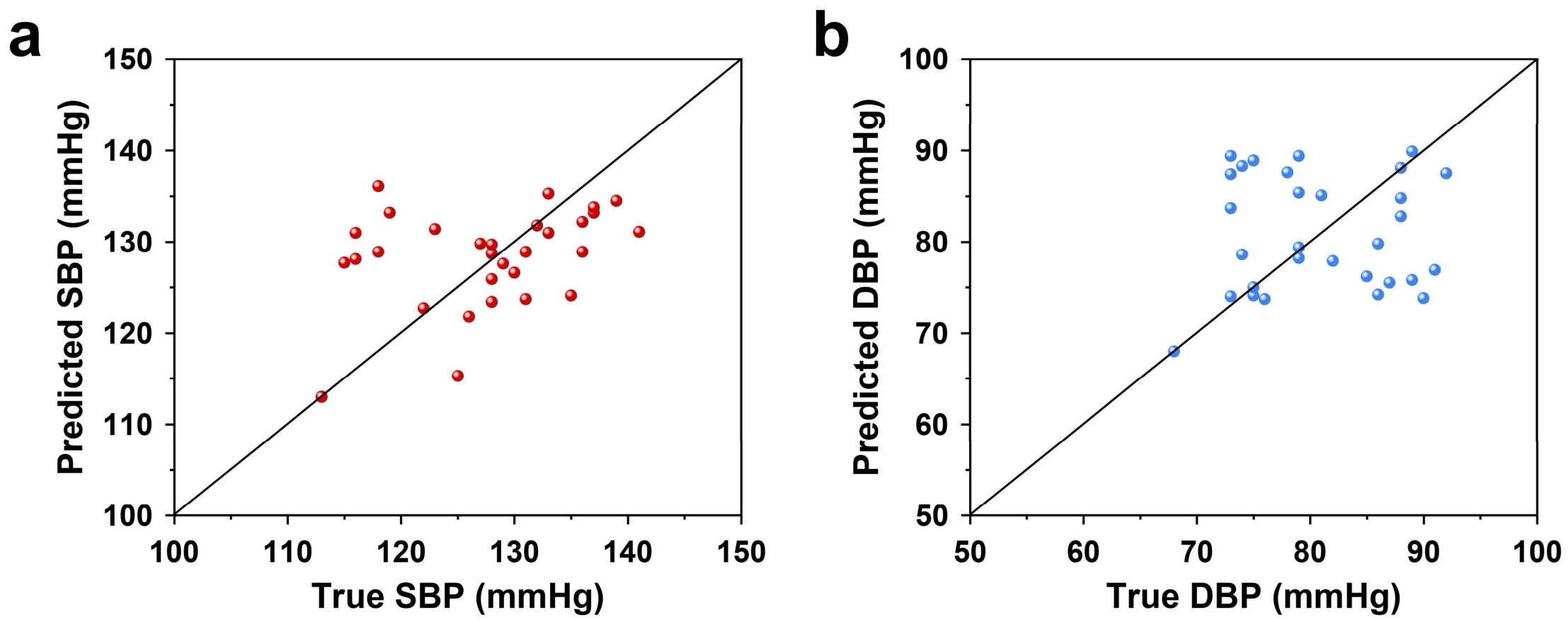

Fig. S20. Scatter plots of model-estimated values for (A) SBP and (B) DBP compared with reference values. Each dot represents a single measurement ($n = 30$). The solid line represents the index line ($y = x$). Points above or below this line indicate overestimation or underestimation.

Table S1. Comparison of mechanical properties with previously reported piezoionic materials

Material	Toughness (MJ m^{-3})	Ultimate tensile stress (MPa)	Elongation at break	Reference
Poly (LiTFSI-TA-MBA)	1.22	0.95	193%	11
PVDF-HFP/IPC	1.4	0.3	520%	15
Crown ether grafted PVA	2.45	1.22	390%	18
PU	9.45	1.66	1201%	24
(PAA-co-PAM)-PVA	2.75	1.55	359%	25
**PVA-PU**	**23.1**	**2.88**	**1510%**	**This work**

Table S2. Comparison of mechanical properties with previously reported high toughness gels.

Material	Elongation at break	Toughness (MJ m^{-3})	Young's modulus (MPa)	Ultimate tensile stress (MPa)	Reference
PVA / CA	520%	22	24.7	17.4	37
PVA / Gly / ChCl	530%	86.8	103.1	40.5	38
PVA / TA-Br / TA / Fe^{3+}	550%	32	3.5	13.8	39
PVA-PU / BMIMTFSI	1510%	23.1	0.3	2.88	This work

Table S3. Comparison of piezoionic properties with previously reported piezoionic materials

	Materials	Voltage	Current	Piezoionic coefficient	Pressure response range	Response time	Recovery time	Detection limit	Ionic conductivity	Current density	Peak Power density
This work	PVA-PU/BMIMTFSI	20 mV	450 nA	1613 nV/Pa	0–500 kPa	96 ms	0.77 s	127 Pa	0.42 S/m	0.5 μA/cm^2	15.2 nW/cm^3
Ref. 40	PAAm-LiCl/PET fiber	30 mV	–	0.043 mV/kPa	0–156.9 kPa	–	–	–	–	65 μA/cm^2	–
Ref. 21	PVDF-HFP/IL/IPC	90 mV	92 nA	–	–	–	–	–	0.3 S/m	–	1.3 μW/cm^3
Ref. 41	PIL(BA-MA)（BMI^+/APS^-）	32.2 mV	0.7 μA	368 nV/Pa	1–210 kPa	20 ms	–		–	–	8.0 nW/cm^3
Ref. 42	PAM /NaCl	~10 mV	~150 nA	1.79 nA/Pa	15–75 kPa	–	–	–	–	–	–
Ref. 43	PVA-CE/NaCl	3.50mV	–	1490 nV/Pa	–	245 ms	388 ms	0.2 Pa	4.29 S/m	–	1.9 μW/cm^3
Ref. 20	pAAm/NaCl	–	–	8 μV/kPa	20–360 kPa	–	–	–	~1 S/m	10 μA/ cm^2	0.85 μW·cm^{-3}

Table S4. The BHS standard

Error	≤ 5mmHg	≤ 10mmHg	≤ 15mmHg
Grade A	60%	85%	95%
Grade B	50%	75%	90%
Grade C	40%	65%	85%
**This work**	**72%**	**90%**	**97%**

Movie S1. Investigation of mobile ion species in the piezoionic ionogel by selective electrode pressing.

Movie S2. Demonstration of a cuffless, noninvasive, and continuous blood pressure monitoring system.

REFERENCES

1. W. Tang, Q. Sun, Z. L. Wang, Self-powered sensing in wearable electronics—A paradigm shift technology. *Chem. Rev.* **123**, 12105–12134 (2023).

2. Q. Wang, H. Guan, C. Wang, P. Lei, H. Sheng, H. Bi, J. Hu, C. Guo, Y. Mao, J. Yuan, M. Shao, Z. Jin, J. Li, W. Lan, A wireless, self-powered smart insole for gait monitoring and recognition via nonlinear synergistic pressure sensing. *Sci. Adv.* **11**, eadu1598 (2025).

3. Z. Lai, J. Xu, C. R. Bowen, S. Zhou, Self-powered and self-sensing devices based on human motion. *Joule* **6**, 1501–1565 (2022).

4. Y. Zhou, X. Xiao, G. Chen, X. Zhao, J. Chen, Self-powered sensing technologies for human Metaverse interfacing. *Joule* **6**, 1381–1389 (2022).

5. M. T. Chorsi, E. J. Curry, H. T. Chorsi, R. Das, J. Baroody, P. K. Purohit, H. Ilies, T. D. Nguyen, Piezoelectric biomaterials for sensors and actuators. *Adv. Mater.* **31**, e1802084 (2019).

6. S. Pan, Z. Zhang, Fundamental theories and basic principles of triboelectric effect: A review. *Friction* **7**, 2–17 (2019).

7. W. Tang, Y. Wang, G. Xiang, X. Zhao, Z. Pan, Y. Wang, Y. Yang, Y. Wang, G. Yuan, Enhanced high-power performance of Fe-doped PZMNZT piezoelectric ceramics. *J. Am. Ceram. Soc.* **106**, 6868–6878 (2023).

8. Y. Yan, L. D. Geng, H. Liu, H. Leng, X. Li, Y. U. Wang, S. Priya, Near-ideal electromechanical coupling in textured piezoelectric ceramics. *Nat. Commun.* **13**, 3565 (2022).

9. X. Zhang, Z. Li, W. Du, Y. Zhao, W. Wang, L. Pang, L. Chen, A. Yu, J. Zhai, Self-powered triboelectric-mechanoluminescent electronic skin for detecting and differentiating multiple mechanical stimuli. *Nano Energy* **96**, 107115 (2022).

10. H. Xiang, L. Peng, Q. Yang, Z. L. Wang, X. Cao, Triboelectric nanogenerator for high-entropy energy, self-powered sensors, and popular education. *Sci. Adv.* **10**, eads2291 (2024).

11. W.-G. Kim, D.-W. Kim, I.-W. Tcho, J.-K. Kim, M.-S. Kim, Y.-K. Choi, Triboelectric nanogenerator: Structure, mechanism, and applications. *ACS Nano* **15**, 258–287 (2021).

12. K. Chen, D. Ho, Piezoionics: Mechanical-to-ionic transduction for sensing, biointerface, and energy harvesting. *Aggregate* **5**, e425 (2024).

13. D. Ho, The piezoionic effect: Biomimetic transduction mechanism for sensing, actuation, interface, and energy harvesting. *ChemElectroChem* **11**, e202300268 (2024).

14. E. K. Boahen, B. Pan, H. Kweon, J. S. Kim, H. Choi, Z. Kong, D. J. Kim, J. Zhu, W. B. Ying, K. J. Lee, D. H. Kim, Ultrafast, autonomous self-healable iontronic skin exhibiting piezo-ionic dynamics. *Nat. Commun.* **13**, 7699 (2022).

15. Z. Fang, L. Wang, C. Liang, J. Qiu, T. Zhang, D. Xu, D. Qi, S. Luan, H. Shi, Bioinspired ionic elastomer with skin-like piezo-ionic dynamics enabled by a self-confined multi-interaction network. *Adv. Funct. Mater.* **35**, 2418583 (2025).

16. J. I. Lee, H. Choi, S. H. Kong, S. Park, D. Park, J. S. Kim, S. H. Kwon, J. Kim, S. H. Choi, S. G. Lee, D. H. Kim, M. S. Kang, Visco-poroelastic electrochemiluminescence skin with piezo-ionic effect. *Adv. Mater.* **33**, 2100321 (2021).

17. Y. Li, H. Yin, C. Jiang, Y. Chen, Q. Li, B. Li, Y. Guo, Rapid fabrication of antifreezing piezoionic hydrogels for fast charging and enhanced energy harvesting frequency via ion-regulating nanoparticles. *Adv. Funct. Mater.* **10**, e15804 (2025).

18. T. Xu, L. Jin, Y. Ao, J. Zhang, Y. Sun, S. Wang, Y. Qu, L. Huang, T. Yang, W. Deng, W. Yang, All-polymer piezo-ionic-electric electronics. *Nat. Commun.* **15**, 10876 (2024).

19. S. Wang, T. Yang, D. Zhang, Q. Hua, Y. Zhao, Unveiling gating behavior in piezoionic effect: Toward neuromimetic tactile sensing. *Adv. Mater.* **36**, e2405391 (2024).

20. Y. Dobashi, D. Yao, Y. Petel, T. N. Nguyen, M. S. Sarwar, Y. Thabet, C. L. W. Ng, E. S. Glitz, G. T. M. Nguyen, C. Plesse, F. Vidal, C. A. Michal, J. D. W. Madden, Piezoionic mechanoreceptors: Force-induced current generation in hydrogels. *Science* **376**, 502–507 (2022).

21. W. Zhu, B. Wu, Z. Lei, P. Wu, Piezoionic elastomers by phase and interface engineering for high-performance energy-harvesting ionotronics. *Adv. Mater.* **36**, e2313127 (2024).

22. J. Xu, Q. Li, D. Ho, A universal framework for determining the effect of operating parameters on piezoionic voltage generation. *Mater. Horiz.* **11**, 5709–5721 (2024).

23. N. Tang, Y. Jiang, K. Wei, Z. Zheng, H. Zhang, J. Hu, Evolutionary reinforcement of polymer networks: A stepwise-enhanced strategy for ultrarobust eutectogels. *Adv. Mater.* **36**, e2309576 (2024).

24. A. Xu, J. Liu, Y. Wang, Z. Xiao, Y. Xie, Leaf-inspired cellulose ionogels under spatial confinement with synergistically enhanced mechanical and sensing performance for flexible sensors. *Adv. Funct. Mater.* **36**, e19010 (2026).

25. K. Yang, B. Li, Z. Ma, J. Xu, D. Wang, Z. Zeng, D. Ho, Ion-selective mobility differential amplifier: Enhancing pressure-induced voltage response in hydrogels. *Angew. Chem. Int. Ed.* **64**, e202415000 (2025).

26. J. Tie, H. Liu, J. Lv, B. Wang, Z. Mao, L. Zhang, Y. Zhong, X. Feng, X. Sui, H. Xu, Multi-responsive, self-healing and adhesive PVA based hydrogels induced by the ultrafast complexation of Fe3+ ions. *Soft Matter* **15**, 7404–7411 (2019).

27. S. Wu, M. Hua, Y. Alsaid, Y. Du, Y. Ma, Y. Zhao, C.-Y. Lo, C. Wang, D. Wu, B. Yao, J. Strzalka, H. Zhou, X. Zhu, X. He, Poly(vinyl alcohol) hydrogels with broad-range tunable mechanical properties via the hofmeister effect. *Adv. Mater.* **33**, e2007829 (2021).

28. M. Hua, S. Wu, Y. Ma, Y. Zhao, Z. Chen, I. Frenkel, J. Strzalka, H. Zhou, X. Zhu, X. He, Strong tough hydrogels via the synergy of freeze-casting and salting out. *Nature* **590**, 594–599 (2021).

29. Y. Jiang, N. Tang, X. Wang, D. Pei, H. Zhang, M.-H. Li, J. Hu, Mechanically robust eutectogels enabled by precisely engineered crystalline domains. *Nat. Commun.* **16**, 7417 (2025).

30. W. Zhao, Y. Zhang, X. Zhao, B. Yu, L. Zhang, S. Ma, F. Zhou, Superior anti-swelling and durably lubricious bio-hydrogels via robust crystalline domain construction for diverse biodevice coating. *Matter* **8**, 102317 (2025).

31. Y. Li, Y. Li, R. Cao, J. Xie, W. Chen, Amplifying the mechanical resilience of chemically cross-linked poly(vinyl alcohol) films with the addition of boric acid. *Macromolecules* **57**, 6321–6332 (2024).

32. S. Yin, Y. Ding, Y. Zhang, C. Zhou, C. Qian, G. Che, C. Zhao, L. Jiang, Superstrong and transparent hydrogels with homogeneous multiple networks. *Macromolecules* **58**, 4357–4364 (2025).

33. L. Xu, Y. Qiao, D. Qiu, Coordinatively stiffen and toughen hydrogels with adaptable crystal-domain cross-linking. *Adv. Mater.* **35**, e2209913 (2023).

34. H. Zhuo, X. Dong, Q. Liu, L. Hong, Z. Zhang, S. Long, W. Zhai, Bamboo-inspired ultra-strong nanofiber-reinforced composite hydrogels. *Nat. Commun.* **16**, 980 (2025).

35. Z. Kong, E. K. Boahen, D. J. Kim, F. Li, J. S. Kim, H. Kweon, S. Y. Kim, H. Choi, J. Zhu, W. Bin Ying, D. H. Kim, Ultrafast underwater self-healing piezo-ionic elastomer via dynamic hydrophobic-hydrolytic domains. *Nat. Commun.* **15**, 2129 (2024).

36. X. Guan, S. Zheng, J. Luo, X. Liu, X. Wang, B. Zhang, Y. Zhu, D. Li, Q. Han, M. Ueda, M. An, Spider webs-inspired aluminum coordination hydrogel piezoionic sensors for tactile nerve systems. *Adv. Funct. Mater.* **35**, 2414016 (2025).

37. Q. Wei, C. Zhang, Y. Quan, H. Shi, Y. Zhang, R. Xu, S. Ma, F. Zhou, Enhancing superlubricity and wear resistance in mechanically robust hydrogel via microliter-scale subsurface-initiated polymer brush grafting. *Angew. Chem. Int. Ed. Engl.* **65**, e24676 (2026).

38. J. Dai, Y. Xue, X. Chen, Z. Cao, L. Wang, J. Zhang, Y. Zhou, Y. Hu, W. Zhou, W. Tang, X.-Y. Kong, B. Tu, J. Liu, K. Xiao, Artificial nerve for neuromodulation based on structured piezoionic hydrogel. *Device* **2**, 100436 (2024).

39. D. Wang, Z. Zeng, Z. Ma, B. Li, J. Shao, K. Yang, D. Ho, Soft self-powered solid-state poly(ionic liquid) piezoionic elastomer sensor for extreme temperature operation. *Chem. Eng. J.* **530**, 173003 (2026).

40. Y. Sun, G. Tian, T. Yang, S. Wang, B. Lan, X. Li, T. Xu, L. Jin, W. Deng, W. Yang, Crosslinking-modulated hydrogel piezoionic sensor for pattern security authentication in human-machine interfaces. *Adv. Funct. Mater.* **35**, 2420187 (2025).

41. S. G. Chrysant, Relatability of blood pressure monitoring with wearable cuffless devices. *Am. J. Cardiol.* **169**, 145–147 (2022).

42. S. Min, J. An, J. H. Lee, J. H. Kim, D. J. Joe, S. H. Eom, C. D. Yoo, H.-S. Ahn, J.-Y. Hwang, S. Xu, J. A. Rogers, K. J. Lee, Wearable blood pressure sensors for cardiovascular monitoring and machine learning algorithms for blood pressure estimation. *Nat. Rev. Cardiol.* **22**, 629–648 (2025).

43. Y. Duan, B. Li, K. Yang, Z. Gong, X. Peng, L. He, D. Ho, Ultrahigh energy and power density in Ni–Zn aqueous battery via superoxide-activated three-electron transfer. *Nano Micro Lett.* **17**, 79 (2024).

44. F. Schrumpf, P. Frenzel, C. Aust, G. Osterhoff, M. Fuchs, Assessment of non-invasive blood pressure prediction from PPG and rPPG signals using deep learning. *Sensors* **21**, 6022 (2021).

45. T. Liu, Y. Mao, H. Dou, W. Zhang, J. Yang, P. Wu, D. Li, X. Mu, Emerging wearable acoustic sensing technologies. *Adv. Sci.* **12**, e2408653 (2025).

46. S. Zhou, G. Park, K. Longardner, M. Lin, B. Qi, X. Yang, X. Gao, H. Huang, X. Chen, Y. Bian, H. Hu, R. S. Wu, W. Yue, M. Li, C. Lu, R. Wang, S. Qin, E. Tasali, T. Karrison, I. Thomas, B. Smarr, E. B. Kistler, B. A. Khiami, I. Litvan, S. Xu, Clinical validation of a wearable ultrasound sensor of blood pressure. *Nat. Biomed. Eng.* **9**, 865–881 (2025).

47. X. Wang, G. Wu, X. Zhang, F. Lv, Z. Yang, X. Nan, Z. Zhang, C. Xue, H. Cheng, L. Gao, Traditional chinese medicine (TCM)-inspired fully printed soft pressure sensor array with

self-adaptive pressurization for highly reliable individualized long-term pulse diagnostics. *Adv. Mater.* **37**, e2410312 (2025).

48. S. Min, D. H. Kim, D. J. Joe, B. W. Kim, Y. H. Jung, J. H. Lee, B.-Y. Lee, I. Doh, J. An, Y.-N. Youn, B. Joung, C. D. Yoo, H.-S. Ahn, K. J. Lee, Clinical validation of a wearable piezoelectric blood-pressure sensor for continuous health monitoring. *Adv. Mater.* **35**, e2301627 (2023).

49. X. Ran, F. Luo, Z. Lin, Z. Zhu, C. Liu, B. Chen, Blood pressure monitoring via double sandwich-structured triboelectric sensors and deep learning models. *Nano Res.* **15**, 5500–5509 (2022).

50. M. Amjad, I. Ahmad, M. Ahmad, P. Wróblewski, P. Kamiński, U. Amjad, Prediction of pile bearing capacity using XGBoost algorithm: Modeling and performance evaluation. *Appl. Sci.* **12**, 2126 (2022).

51. J. Li, H. Jia, J. Zhou, X. Huang, L. Xu, S. Jia, Z. Gao, K. Yao, D. Li, B. Zhang, Y. Liu, Y. Huang, Y. Hu, G. Zhao, Z. Xu, J. Li, C. K. Yiu, Y. Gao, M. Wu, Y. Jiao, Q. Zhang, X. Tai, R. H. Chan, Y. Zhang, X. Ma, X. Yu, Thin, soft, wearable system for continuous wireless monitoring of artery blood pressure. *Nat. Commun.* **14**, 5009 (2023).

52. M. Frisch, G. W. Trucks, H. B. Schlegel, G. E. Scuseria, M. A. Robb, J. R. Cheeseman, G. Scalmani, V. Barone, B. Mennucci, G. A. Petersson, H. Nakatsuji, M. Caricato, X. Li, H. P. Hratchian, A. F. Izmaylov, J. Bloino, G. Zheng, J. L. Sonnenberg, M. Hada, M. Ehara, K. Toyota, R. Fukuda, J. Hasegawa, M. Ishida, T. Nakajima, Y. Honda, O. Kitao, H. Nakai, T. Vreven, J. A. Montgomery, Jr., J. E. Peralta, F. Ogliaro, M. Bearpark, J. J. Heyd, E. Brothers, K. N. Kudin, V. N. Staroverov, T. Keith, R. Kobayashi, J. Normand, K. Raghavachari, A. Rendell, J. C. Burant, S. S. Iyengar, J. Tomasi, M. Cossi, N. Rega, J. M. Millam, M. Klene, J. E. Knox, J. B. Cross, V. Bakken, C. Adamo, J. Jaramillo, R. Gomperts, R. E. Stratmann, O. Yazyev, A. J. Austin, R. Cammi, C. Pomelli, J. W. Ochterski, R. L. Martin, K. Morokuma, V. G. Zakrzewski, G. A. Voth, P. Salvador, J. J. Dannenberg, S. Dapprich, A. D. Daniels, Ö. Farkas, J. B. Foresman, J. V. Ortiz, J. Cioslowski, and D. J. Fox, Gaussian 09, Revision D.01 (Gaussian Inc., 2009).

53. S. Grimme, J. Antony, S. Ehrlich, H. Krieg, A consistent and accurate ab initio parametrization of density functional dispersion correction (DFT-D) for the 94 elements H-Pu. *J. Chem. Phys.* **132**, 154104 (2010).

54. F. Weigend, R. Ahlrichs, Balanced basis sets of split valence, triple zeta valence and quadruple zeta valence quality for H to Rn: Design and assessment of accuracy. *Phys. Chem. Chem. Phys.* **7**, 3297–3305 (2005).

55. T. Lu, F. Chen, Multiwfn: A multifunctional wavefunction analyzer. *J. Comput. Chem.* **33**, 580–592 (2012).

56. T. Lu, A comprehensive electron wavefunction analysis toolbox for chemists, Multiwfn. *J. Chem. Phys.* **161**, 082503 (2024).

57. B. Hess, C. Kutzner, D. van der Spoel, E. Lindahl, GROMACS 4: Algorithms for highly efficient, load-balanced, and scalable molecular simulation. *J. Chem. Theory Comput.* **4**, 435–447 (2008).

58. U. Essmann, L. Perera, M. L. Berkowitz, T. Darden, H. Lee, L. G. Pedersen, A smooth particle mesh Ewald method. *J. Chem. Phys.* **103**, 8577–8593 (1995).

59. B. Hess, H. Bekker, H. J. C. Berendsen, J. G. E. M. Fraaije, LINCS: A linear constraint solver for molecular simulations. *J. Comput. Chem.* **18**, 1463–1472 (1997).

60. G. E. Karniadakis, I. G. Kevrekidis, L. Lu, P. Perdikaris, S. Wang, L. Yang, Physics-informed machine learning. *Nat. Rev. Phys.* **3**, 422–440 (2021).

61. S. Lee, S. Lee, W. Jang, J. Kim, D. K. Yon, J. Lee, Weighted iterative complex demodulation for high-resolution instantaneous frequencies in low-frequency PPG signals from wearable devices. *IEEE J. Biomed. Health Inform.* **29**, 7149–7160 (2025).

62. T. Chen, C. Guestrin, "XGBoost: A scalable tree boosting system," in *Proceedings of the 22nd ACM SIGKDD International Conference on Knowledge Discovery and Data* (ACM, 2016), vol. 16, pp. 785–794.
